# TLR-2/TLR-4 TREM-1 Signaling Pathway Is Dispensable in Inflammatory Myeloid Cells during Sterile Kidney Injury

**DOI:** 10.1371/journal.pone.0068640

**Published:** 2013-07-03

**Authors:** Gabriela Campanholle, Kristen Mittelsteadt, Shunsaku Nakagawa, Akio Kobayashi, Shuei-Liong Lin, Sina A. Gharib, Jay W. Heinecke, Jessica A. Hamerman, William A. Altemeier, Jeremy S. Duffield

**Affiliations:** 1 Division of Nephrology, University of Washington, Seattle, Washington, United States of America; 2 Center for Lung Biology, Department of Medicine & Pathology, University of Washington, Seattle, Washington, United States of America; 3 Institute of Stem Cell & Regenerative Medicine, University of Washington, Seattle, Washington, United States of America; 4 Diabetes and Obesity Center of Excellence, University of Washington, Seattle, Washington, United States of America; 5 Brigham & Women’s Hospital & Harvard Medical School, Boston, Massachusetts, United States of America; 6 Department of Internal Medicine, National Taiwan University Hospital, Taipei, Taiwan; 7 Benaroya Research Institute at Virginia Mason, Seattle, Washington, United States of America; DRFZ, Germany

## Abstract

Inflammatory macrophages are abundant in kidney disease, stimulating repair, or driving chronic inflammation and fibrosis. Damage associated molecules (DAMPs), released from injured cells engage pattern recognition receptors (PRRs) on macrophages, contributing to activation. Understanding mechanisms of macrophage activation during kidney injury may lead to strategies to alleviate chronic disease. We identified Triggering-Receptor-in-Myeloid-cells (TREM)-1, a regulator of TLR signaling, as highly upregulated in kidney inflammatory macrophages and tested the roles of these receptors in macrophage activation and kidney disease. Kidney DAMPs activated macrophages *in vitro,* independently of TREM-1, but partially dependent on TLR-2/−4, MyD88. In two models of progressive interstitial kidney disease, TREM-1 blockade had no impact on disease or macrophage activation *in vivo*, but TLR-2/−4, or MyD88 deficiency was anti-inflammatory and anti-fibrotic. When MyD88 was mutated only in the myeloid lineage, however, there was no bearing on macrophage activation or disease progression. Instead, TLR-2/−4 or MyD88 deficiency reduced activation of mesenchyme lineage cells resulting in reduced inflammation and fibrosis, indicating that these pathways play dominant roles in activation of myofibroblasts but not macrophages. To conclude, TREM-1, TLR2/4 and MyD88 signaling pathways are redundant in myeloid cell activation in kidney injury, but the latter appear to regulate activation of mesenchymal cells.

## Introduction

Inflammatory monocytes which are recruited to sites of tissue injury and differentiate into tissue effector macrophages have been shown to play important roles in the progression of chronic kidney diseases and the resolution of acute kidney injury [1], [2]. We previously showed that in mouse models of progressive kidney injury, three subpopulations of macrophages can be discerned in the kidney parenchyma defined by the cell surface marker Ly6C [3]. The Ly6C^high^ population was activated similarly to the M1 activation defined *in vitro,* and the Ly6C^low^ subpopulation bore some similarities to M2a macrophages with the hallmarks of a profibrotic population. We also showed that Ly6C^high^ macrophages differentiate into Ly6C^low^ macrophages [3]. Overall macrophages in this model promote injury and fibrosis. Since all populations of macrophages were activated we hypothesized that danger associated molecular patterns (DAMPs) may play important roles in their activation and that specific pattern recognition receptors (PRRs) may regulate the response of macrophages to DAMPs.

Identification of injury molecules and receptors that activate monocytes when they enter the injured kidney is critical to the development of new treatments focused on macrophages. Increasing evidence points to DAMPs, released from injured parenchymal cells, as critical factors that contribute to the pro-inflammatory phenotype in the injured kidney via PRR-binding and subsequent activation of NF-κB, MAPK and inflammasome signaling [4], [5]. The PRRs that have been most intensely investigated as receptors for DAMPs are the Toll-Like Receptors (TLRs). TLRs are germline encoded transmembrane receptors that recognize pathogen-associated molecular patterns and initiate an intracellular signaling cascade leading ultimately to an inflammatory response. All of the TLRs, with the exception of TLR-3, require the MyD88 adapter protein for maximal response. A number of DAMPs have been identified as ligands for different TLRs, including mitochondrial DNA (TLR-9), histones (TLR-4), hyaluronan fragments (TLR-2 and 4), high mobility group box -1 (TLR-4), and several heat shock proteins (TLR-2 and 4) [6]–[12]. One hypothesis is that the array of DAMPs and associated bound molecules to which monocytes are exposed, determines the state of activation of the myeloid cells.

Triggering receptors expressed on myeloid cell (TREM) family are a cell surface Immunoglobulin domain receptor family restricted to myeloid lineage cells. The TREM family function as modulators of cellular response, regulating positively and negatively the activation of myeloid cells during inflammation. The majority of TREM family members lack cytoplasm signaling motif but associate with an ITAM containing signaling adaptor protein, DAP12 which can recruit activating kinases including Syk. TREM-1 was first characterized in infections, was highly upregulated and has been implicated as an amplifier of inflammation [13] [14]–[17], functioning as an important co-activator of the TLR [13], [17], [18] and NOD Like Receptor (NLR) [19], [20] signaling pathways. Several recent studies suggested that TREM-1 may be an important and targetable effector molecule not only in infections but also in sterile inflammation [21] in pancreas [22], [23], joints [24], gut [25], [26] and eyes [4], [27]. In addition, TREM-1 is cleaved, and soluble TREM-1 is readily detected in biological fluids of patients suffering from a variety of diseases [28], suggesting a possible role as a decoy receptor that competes for putative ligands and negatively regulates TREM-1 pathway activation.

In these studies we identified TREM-1 as a highly expressed receptor in macrophages during sterile kidney injury. Furthermore, we investigated the role of TREM-1 and the related TLR receptor signaling pathways in macrophage activation and disease progression in the kidney.

## Materials and Methods

### Animals

*CsfR1-iCre* [29] (FVB) mice were bred with *Myd88^fl/fl^* mice (C57BL/6) (Jackson Laboratories) and the F2 generation was used for the experiments. *Tlr2^−/−^, Tlr4^−/−^, Tlr2-4^−/−^* and *Myd88^−/−^* (C57BL/6) mice were previously reported [30]. *Dap12^−/−^* mice were previously reported [31], [32]. All experiments were performed under a protocol approved by the Department of Comparative Medicine, University of Washington (Permit number 4244-01). All surgery was performed under ketamine and xylazine anesthesia and all efforts were made to minimize suffering.

### Mouse Model of Kidney Injury with Fibrosis

Mice were anesthetized with ketamine/xylazine (100/10 mg/kg i.p.) and Unilateral Ureteral Obstruction (UUO) or unilateral Ischemia and Reperfusion Injury (U-IRI) were performed in adult (8–12wk) mice as previously described [33]. In the U-IRI model, left kidney was clamped for 40 minutes (females) at 36.8–37.3°C core temperature. For the TREM-1 experiments, mice received daily i.p. injections of 40 µg of purified TREM1-Fc or hIgG1, as control, diluted in PBS, starting at the day of surgery until sacrifice at day 5.

### Tissue Preparation and Histology

Mouse tissues were prepared and stained as previously described [33], [34]. Primary antibodies against the following proteins were used for immunolabeling: CD11b (e-bioscience 1∶200), Ly6C (e-bioscience 1∶200), anti-TREM-1 (R&D 1∶200), F4/80 (Invitrogen 1∶200) and αSMA (Sigma 1∶400). Slides were incubated with Fluorescence (Cy3 or FITC)-conjugated secondary antibodies (1∶400–1∶800, Jackson ImmunoResearch), mounted with Vectashield/DAPI, and images were captured using a Nikon TiE Inverted Widefield Fluorescence microscope at the Lynn and Mike Garvey Cell Imaging Core at Institute for Stem Cell and Regenerative Medicine of University of Washington. For morphometric analysis of collagen fibril staining, deparaffinized sections (3 µm) were stained with 0.1% picrosirius red [35], [36]. Area of positive fluorescence/stain in 200× magnification of 10 randomly selected images per mouse were quantified using Image J software (http://rsbweb.nih.gov/ij/) [34], [37].

### Q-PCR

RNA was isolated from kidney tissue samples using TRIzol (Invitrogen) according to standard protocol. First-strand cDNA was synthesized using the iScript kit (Bio-rad). Real-time PCR was performed using iTaq SYBR green supermix with ROX (Bio-rad) and 7900HT ABI detection system (Applied Biosystems). Target genes were normalized by Hypoxanthine phosphoribosyltransferase (*HPRT*) expression. The mRNA expression was calculated using the 2^-ΔΔCt^ method and expressed as an n-fold difference relative to the control group.

### Kidney Danger Associated Molecular Pattern Preparation

Kidneys were collected from normal mice (control), or day 5 after UUO. Under sterile conditions kidney vasculature was flushed with ice cold PBS. Under sterile conditions kidneys were decapsulated, minced and incubated with 2ml of LIBERASE TL (0.2mg/ml in DMEM/F12, Roche) and digested by shaking vigorously in a water-bath (37°C, 30min). Five ml of PBS was added, then the single cell preparation filtered through a 40 µm Cell Strainer. The filtrate was centrifuged (2000rpm, 5min at 4°C) to pellet any cellular debris and the cell-free supernatant was filtered using 0.2 µm low protein binding syringe filter. Polymyxin B beads (Sigma P1411), 20 µl, were added to the supernatant and incubated at 4°C for 30 minutes to remove any contamination by endotoxin. Supernatant containing DAMPs centrifuged (2000rpm, 5min, 4°C) to remove beads, and aliquots were stored at −80°C until use.

### Bone Marrow Macrophage and Pericyte Isolation

BMDMφ were generated by flushing femurs with DMEM/F12 as previously described [29]. BMDMφ was cultured for 7 days in macrophage medium (DMEM/F12 medium (Cellgro), containing 10% FBS (Invitrogen), 1% Penicillin/Streptomycin (Cellgro), 20% L929 conditioned media containing M-CSF and stimulated on day 8. Pericytes from normal kidney from C57BL/6 wild-type, *Tlr2–4^−/−^* and *Myd88^−/−^* mice were isolated using MACS (Miltenyi Biotech) and rabbit polyclonal anti-PDGFRβ antibody as detailed previously [37].

### Bone Marrow Macrophage and Pericyte Stimulation

BMDMφ were incubated in 24well/plate (0.5×10^6^ cells/well) with 1ml of DMEM/F12 serum-free media per well, containing 100 µl of crude DAMPS from injured or normal kidney, or 100 µl of Liberase mix as control. To activate TREM-1 in BMDMφ, anti-TREM-1 antibodies (R&D) at 1 or 2 µg/ml or isotype IgG control (R&D) were applied. To block TREM-1 activity, purified TREM1-Fc or human IgG (Sigma) was applied at 1 or 3 µg/ml. Pericytes between passages 2 and 5 were stimulated in 12-well plates pre-coated with gelatin when 60–80% confluent. The concentrations of IL-6 and MCP-1 in the supernatants of primary pericytes were determined using enzyme-linked immunosorbent assay kits (BioLegend) according to the vendor’s protocols.

### TREM1-Fc Generation and Purification

*Trem1* ORF was cloned from cDNA from LPS activated BMDMφs. To generate a soluble TREM1-Fc fusion protein, the cDNA encoding the extracellular region of *Trem1* (position 57 to position 659 in the gene; NCBI Reference NM_021406.5) was amplified by PCR, digested and subcloned, in frame, into the multiple cloning site of (pFUSE-hIgG1-Fc1, InvivoGen) containing the Fc region of human IgG_1_ (hinge, CH2, CH3), and confirmed by sequencing. Plasmid DNA was obtained by Maxiprep (Qiagen) and transfected using lipofectamine LTX reagent (Invitrogen) to approximately 60% confluent 293T cells in 75 flasks that were seeded the day before. After 24h of transfection, cells were washed with PBS, and incubated with DMEM/F12 serum-free media for 4 days. Supernatant was collected, centrifuged, filtered and TREM1-Fc protein was purified by affinity to Protein A - Sepharose beads column (Invitrogen) using standard methods described [38]. After elution, TREM1-Fc was dialyzed for 16h at 4°C, using 20.000 MWCO cassettes (Thermo Scientific) to exchange the current elution buffer to PBS. Presence and purity of TREM1-Fc protein was confirmed by SDS PAGE gel stained with Gel Code Blue Safe Protein Stain (Thermo Scientific) and western blot using anti-TREM-1 antibody (R&D).

### SDS PAGE and Western Blotting

Kidneys or BMDMφs lysates were separated on 10% SDS-PAGE gel (Bio-rad) then semi-dry transferred to an Immobilon PVDF membrane as described [37], [39]. After blocking, membranes were incubated overnight with primary antibodies, anti-TREM-1 (R&D, 1∶1000), anti-MyD88 (ProSci, 1∶500), β-Actin (Santa Cruz, 1∶1000), anti-HMGB1 (BioLegend 1∶1000), anti-mouse IgG (Jackson ImmunoResearch, 1∶2500). Horseradish peroxidase-conjugated secondary antibodies (Pierce) were applied and enhanced chemiluminescence (Thermo Scientific) was used to detect proteins, and images collected by FluorChemQ machine (Alpha Innotech Corporation).

### Statistical Analysis

Statistical evaluation was carried out using the One Way Analysis of Variance (ANOVA) followed by Tukey post-test using GraphPad Prism (GraphPad Software). A p value <0.05 was considered to be significant. Error bars indicate standard error of mean.

## Results

### TREMs are Highly Upregulated in Mouse Models of Chronic Kidney Injury

**Figure 1:**
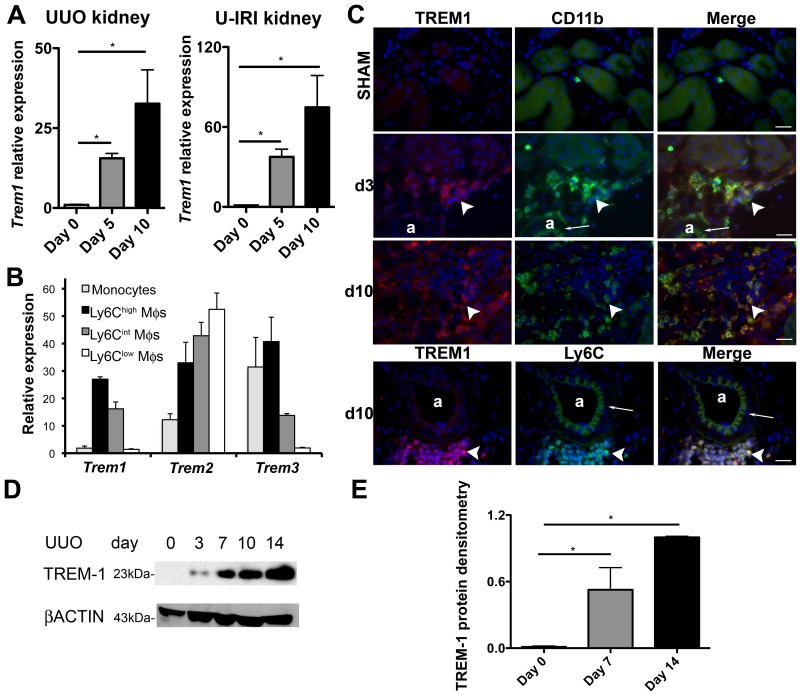
TREMs are highly expressed in macrophages during kidney injury. (**A**) Q-PCR for *Trem1* expression in whole kidney 0, 5 and 10 days after UUO and U-IRI. (**B**) Q-PCR for TREM family transcript expression in blood monocytes, and different sub-populations of kidney macrophages purified at day 5 after UUO. (**C**) Fluorescence images showing CD11b (green), Ly6C (green) and TREM-1 (red) expression in tissue sections from control kidney (sham), day 3 and 10 after UUO (a, arteriole; Bar = 25 µm; arrowhead shows interstitial CD11b+ and TREM-1+ cells; arrow shows autofluorescent arteriole internal elastic lamina). (**D**) Western blot of whole kidney lysates detecting TREM-1 (23kD) and β-Actin (43kD), 0, 3, 7, 10 and 14 days after UUO. (**E**) TREM-1 protein densitometry normalized to endogenous control β-Actin. (n = 3–5/group, 3 independent experiments; *P<0.05).

We purified macrophage subpopulations, discriminated by the subpopulation marker, Ly6C, from kidneys 5 days after inducing progressive interstitial kidney disease by unilateral ureteral obstruction (UUO) using flow sorting **(see Methods S1)**. Neutrophils and NK cells were excluded **(Fig. S1)**. The transcriptome of those macrophage subpopulations was interrogated by microarray to identify regulated genes that separate M1 (Ly6C^high^) from M2a (Ly6C^low^) type macrophages *in vivo*. Using a highly stringent algorithm to identify regulated genes (**Fig. S2**), we discovered that the majority of genes were down regulated from comparing Ly6C^+^ to Ly6C^low^ cells and that the genes could be clustered in terms of biological processes, including immune response, response to stimulus, migration and chemotaxis. Among the immune response genes were many associated with either pattern recognition or activation (*Trem1, Trem3, Pglyrp1, Clec4d, Clec4e, Tsg6 and Schlafen4, S100a8* and *S100a9*), strongly suggesting that Ly6C^low^ macrophages down-regulate activation pathways compared with Ly6C^+^ macrophages. Since TREM-1 has been shown to be an important co-activator of macrophages in inflammatory diseases, we investigated the function of TREM-1 further in sterile kidney injury. To validate the transcriptional analysis we quantified transcripts for the TREM family by quantitative RT-PCR (Q-PCR) of whole kidney or purified macrophage subpopulations or autologous blood monocytes during the evolution of the UUO model of progressive kidney injury (**Fig. 1A–B**). *Trem1* was highly upregulated in whole kidney, 5 and 10 days after UUO, as well as in a second model of kidney injury with chronic inflammation and fibrosis, unilateral ischemia and reperfusion injury (U-IRI) **(** **Fig. 1A** **)**. In the UUO model, *Trem1* was particularly upregulated in Ly6C^high^ and intermediate macrophages purified from the kidney (**Fig. 1B**). *Trem3* expression mirrored the regulation pattern of *Trem1* except that *Trem3* was highly expressed by blood monocytes (**Fig. 1B**). *Trem2* was also expressed by monocytes, upregulated in Ly6C^high^ macrophages, but unlike *Trem1* and *Trem3*, was further upregulated in Ly6C^low^ macrophages (**Fig. 1B**). Similarly to transcript levels, TREM-1 protein was not detected in normal kidney but highly upregulated during the progression of the UUO model (**Fig. 1D–E**), and its expression was restricted to CD11b+ and Ly6C+ cells (**Fig. 1C**).

### TLR-2 and TLR-4 but not TREM-1, Regulate Activation of Macrophages *in vitro* by Kidney DAMPs

**Figure 2:**
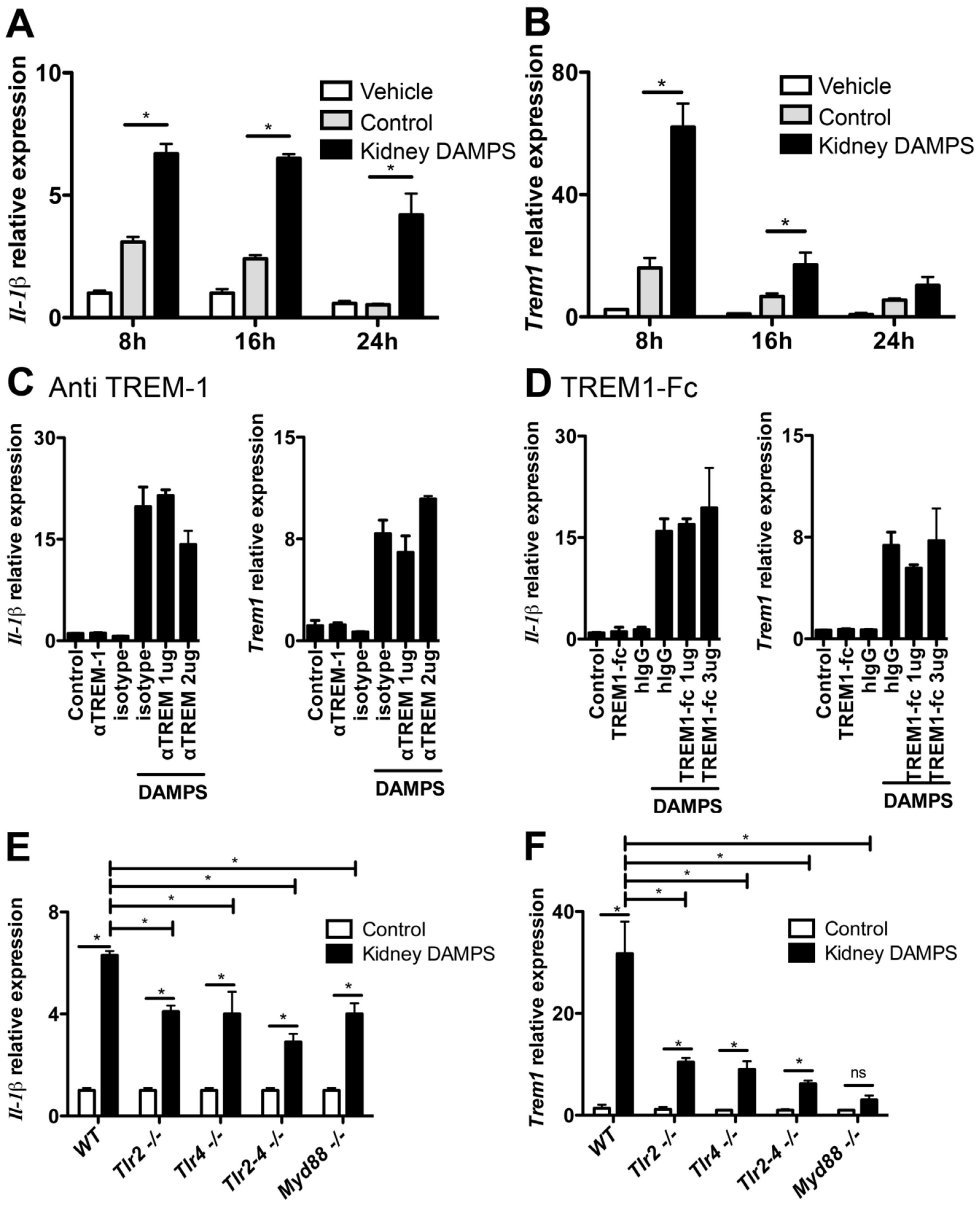
TLR-2 and TLR-4 but not TREM-1, regulate activation of macrophages *in vitro* by kidney DAMPs. **(A–B)** **** Q-PCR showing *Il-1β* and *Trem1* expression in BMDMφ stimulated for 8, 16 or 24h with soluble factors prepared from normal kidney (control) or disease kidney (kidney DAMPs). **(C–D)** Graphs showing *Il-1β* and *Trem1* expression by Q–PCR, 16h after BMDMφ were stimulated with kidney DAMPs and (**C**) activating anti-TREM-1 antibodies, or (**D**) TREM1-Fc, which blocks TREM-1 receptor by competing for ligands. (**E–F**) Q-PCR showing (**E**) *Il-1β* and (**F**) *Trem1* expression in BMDMφ isolated from WT, *Tlr2^−/−^, Tlr4^−/−^, Tlr2–4^−/−^,* and *Myd88^−/−^* mice stimulated with kidney DAMPs for 16h. Q-PCR results were normalized to their respective control group. (*P<0.05, n = 5–7/group, 3 independent experiments; ns, p is not significant).

To study the signaling pathways in DAMP-mediated activation of macrophages in kidney injury and the role of TREM-1 and TLRs in this activation, we separated extracellular soluble factors from injured kidney (kidney DAMPs) or normal kidney (control) and applied these factors to primary cultures of quiescent bone marrow derived macrophages (BMDMφs) as a model of macrophage activation *in vitro*. Kidney DAMPs specifically stimulated *Il-1β* expression, an effect lasting 24h (**Fig. 2A**). Kidney DAMPs also activated *Trem1* expression highly at 8h, but this response returned to baseline at 24h, a finding suggestive that TREM-1 may play a role in enhancing DAMP responses in macrophages and consistent with the findings that kidney macrophages are activated and produce TREM-1 (**Fig. 2B**). Kidney DAMPs did not induce *Tnf-a* production (not shown), similarly to our previous studies which showed kidney macrophages did not show *Tnf-a* activation [3]. Pre-incubation of BMDMφs with IFNγ for 8h markedly augmented subsequent kidney DAMP responses (**Fig. S3A**) suggesting either that cells have been primed by IFNγ to respond more strongly to DAMPs, similar to the enhancement during TLR agonist stimulation, or, alternatively that IFNγ upregulates DAMP receptors. To explore the nature of kidney DAMPs further, the crude kidney DAMPs were separated by SDS PAGE and proteins detected by Coomassie blue stain (**Fig. S3B) (see Methods S1)**. Although, multiple protein bands were visualized in the normal kidney preparation (control), several bands appeared in the DAMPs preparation only suggesting these might be candidate protein DAMP molecules in the soluble preparation (**Fig. S3B**). High-mobility group protein B1 (HMGB1), which has been previously described to be a ligand for TREM-1 [40], [41], was one such DAMP molecule found in abundance in kidney DAMPs **(Fig. S3C)**. Boiling almost completely attenuated biological activity of kidney DAMPs **(Fig. S3D)**. When kidney DAMPs were exposed to trypsin or pronase, however, there was no impact on DAMPs activity, suggesting these DAMP factors specific to macrophages are either resistant to degradation or are non-proteinaceous **(Fig. S3E)**.

We hypothesized that cell surface TREM-1 may function as a co-activator of macrophages during activation by kidney DAMPs. We cloned mouse *Trem1*, and generated an Fc-fusion protein with the *Trem1* ectodomain and the human IgG_1_ Fc domain to use as a decoy receptor to block TREM-1 activation (**Fig. S4A**), a method used successfully by others, such as in attenuating macrophage activation by LPS [13], [17]. To validate our protein, we stimulated BMDMφs with LPS and treated with TREM1-Fc or anti-TREM-1 antibodies, which triggers TREM-1 cell surface clustering and activates TREM-1 signaling. TREM1-Fc abrogated macrophage activation by LPS, and anti-TREM-1 amplified this activation **(Fig. S4B–C).** We then investigated the role of TREM-1 in BMDMφ-activation by kidney DAMPs. In contrast to the results observed with LPS, activation of surface TREM-1 by anti-TREM-1 antibodies did not augment DAMP-mediated activation of macrophages *in vitro* (**Fig. 2C**), and purified TREM1-Fc did not significantly inhibit DAMP-mediated activation of macrophages *in vitro* (**Fig. 2D**). Because TREM-1 is only expressed after exposure to DAMPs, we also pre-activated macrophages with kidney DAMPs, and subsequently blocked DAMP-mediated activation with TREM1-Fc or activated with anti-TREM-1, but this experiment also provided no evidence of inhibition or amplification of activation (**Fig. S4D**). In addition, we pre-incubated kidney DAMPs overnight with TREM1-Fc conjugated beads. TREM-1 bound DAMPs were then separated by centrifugation and the remaining supernatant was applied to macrophages. Compared with controls, TREM1-Fc adsorption did not attenuate DAMP activity (**Fig. S4E**). Furthermore, we tested whether DAMPs could activate DAP12 by signaling through membrane bound TREM-1. We stably expressed a fusion protein of TREM-1 with DAP12 in NFAT-Lacz reporter cells (BWZ TREM1/DAP12) **(see Methods S1)**. Signaling via DAP12 activates the NFAT promoter driving β-galactosidase production, which can be detected by a colorimetric assay. Using this cell line, anti-TREM-1 antibodies in suspension or coated to a plate robustly activated DAP12 signaling and production of β-galactosidase (**Fig. S4F–G**). However, kidney DAMPs either coated to plates or in suspension did not activate LacZ, and therefore DAP12, in these reporter cells, suggesting TREM-1 is not a major target for endogenous activators of innate immune responses. Finally, to test whether TREM-1 may have ligands on dying epithelial cells, the binding capacity of TREM1-Fc to apoptotic kidney proximal epithelial cells (LLC-PK1) was evaluated, but no differences were seen compared to healthy epithelial cells (data not shown) [42].

Because TREM-1 is a regulator of TLR signaling, we next investigated whether TLR-2, TLR-4 and MyD88 signaling pathways played any role in DAMP-mediated activation. Using macrophages deficient in TLR-2, TLR-4, TLR-2 and 4 or MyD88, we assessed their responsiveness to kidney DAMPs. Both Toll like single receptor deficiency in macrophages attenuated the production of *Il-1β* in response to DAMPs, but this inhibition was not enhanced significantly in macrophages lacking both receptors, suggesting they have overlapping specificities for DAMP activity (**Fig. 2E**). MyD88 deficient macrophages were also hypo-responsive to kidney DAMPs (**Fig. 2E**). The hypo-responsiveness was most strikingly seen in terms of *Trem1* transcript induction **(** **Fig. 2F** **)**. Collectively these findings suggest TREM-1 does not play a role in macrophage activation *in vitro* by DAMPs, and TLR-2, TLR-4 and the MyD88 signaling pathway are activated by kidney DAMPs, but that other signaling pathways are also responsible for activation.

### Circulating TREM1-Fc does not Prevent Macrophage Activation, Injury and Fibrosis in Models of Kidney Disease

**Figure 3:**
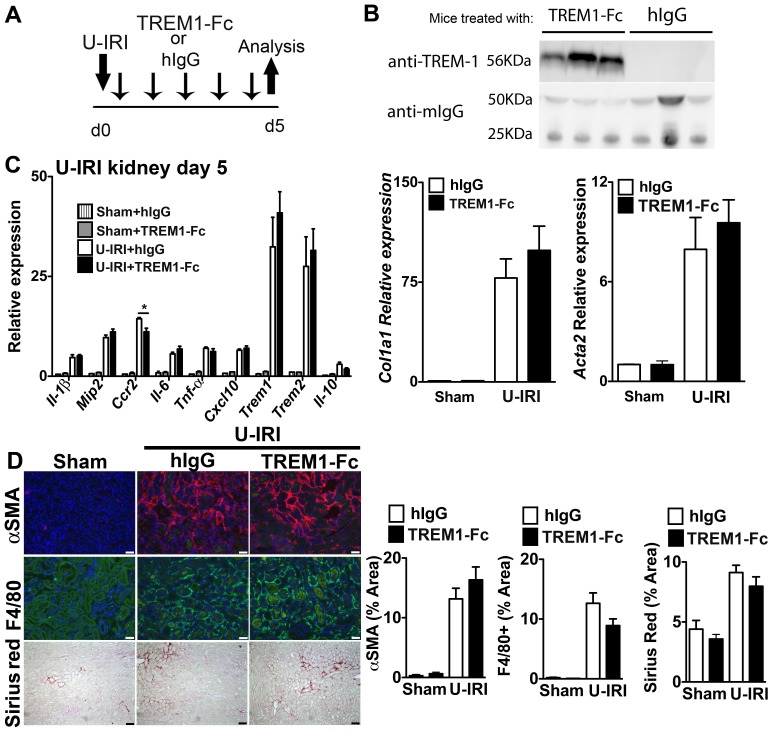
Treatment with soluble TREM1-Fc does not prevent macrophage activation, injury and fibrosis in sterile kidney injury. (**A**) Schema showing experimental design. Mice were subjected to unilateral ischemia and reperfusion injury (U-IRI) and treated daily with 40 µg/mouse of TREM1-Fc or hIgG, as control. (**B**) Western blot showing presence of TREM1-Fc (approximately 56kD) in 2 µl of plasma collected at day 2 from mice treated daily with 40 µg of TREM1-Fc. Anti-mouse IgG was used as endogenous control. (**C**) Q-PCR for different inflammatory transcripts (left) or pro-fibrotic transcripts, Collagen1a1 *(Col1a1)* and alpha smooth muscle actin (*Acta2*), from whole kidney day 5 after U-IRI. (**D**) Representative images (left) and quantitative graphs (right) showing+F4/80 cells (green),+αSMA (red) or collagen deposition (Sirius Red staining) day 5 after U-IRI. (*P<0.05, n = 5–7/group, 3 independent experiments; Bar marker = 50 µm; Q-PCR results were normalized to sham +hIgG).

Our studies indicated that TREM-1 did not play a functional role in BMDMφ activation *in vitro*. To test whether this translated to *in vivo* kidney disease, we induced two different models of kidney injury in mice, UUO and U-IRI, which are characterized by progressive interstitial inflammation and fibrosis [35], and administered TREM1-Fc daily by i.p. injections at a dose of 40 µg/mouse, to give a predicted ECF volume concentration of 4 µg/ml or control human IgG_1_ (**Fig. 3A**). On d2 of the experiment, 2 µl of venous blood was assessed for the presence of TREM1-Fc, which was abundant in mice receiving TREM1-Fc injections but not in mice receiving hIgG injections **(** **Fig. 3B** **).** In both models, whole kidney analysis of macrophage activation genes indicated that TREM1-Fc had no clear impact on macrophage activation (**Fig. 3C, S** **5A**). Because both of these models result in a fibrogenic process, we also evaluated *Col1a1* and *Acta2* transcripts, as indicators of myofibroblast activation and fibrogenesis finding **no** differences (**Fig. 3C, S** **5A**). TREM1-Fc administration had no impact on the extent of macrophage recruitment to the kidney, as well as the extent of αSMA+ myofibroblasts and collagen deposition **(** **Fig. 3D, S** **5B)**. Since TREM-1 signals via the co-receptor DAP12 we evaluated the effect of DAP12 deficiency on the extent of U-IRI kidney disease. Consistent with our observations with administration of TREM1-Fc in vivo, *Dap12^−/−^* mice had similar disease severity compared with strain-matched controls (**Table S1**).

### The TLR-2, TLR-4 and MyD88 Pathways Play a Role in Inflammation and Fibrosis in the U-IRI Model of Sterile Kidney Injury

**Figure 4:**
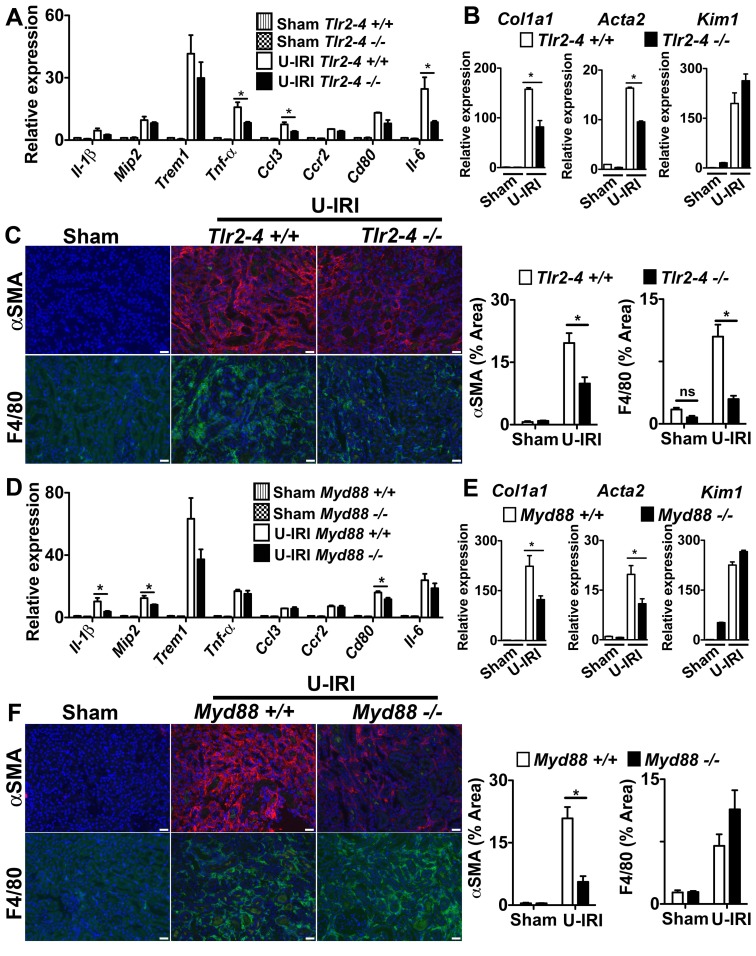
The TLR-2/TLR-4/MyD88 pathways play a role in fibrosis in the U-IRI model of sterile kidney injury. **(A–C)** *Tlr2–4^−/−^* **(D–F)** *Myd88^−/−^* mice or respective controls were subjected to U-IRI and kidney harvested for tissue analysis 5 days later. Q-PCR **(A,D)** for different inflammatory transcripts, **(B,E)** pro-fibrotic transcripts, collagen1a1 (*col1a1*) and alpha smooth muscle actin (*Acta2*), and the tubule injury marker, kidney injury molecule-1 (*Kim-1*) from whole kidney day 5 after U-IRI. **(C,F)** Representative fluorescent images (left) and quantitative graphs (right) showing+αSMA (red) cells and+F4/80 cells (green). (*P<0.05, n = 5–7/group, 3 independent experiments; ns, p is not significant; Bar = 50 µm; Q-PCR results were normalized to wild type control).

The observations from *in vitro* studies (**Fig. 2**) suggested that TLR-2, TLR-4 and MyD88 might be partially responsible for macrophage activation *in vivo*. To explore this possibility further, we studied the progression of two models of kidney disease in mice deficient in TLR-2, TLR-4 and MyD88 (**Fig. 4** **; S6**). Mice lacking MyD88 or both TLRs showed evidence of significant reduction in pro-inflammatory cytokine production in the U-IRI model (**Fig. 4A,D**), but the pattern of reduction was distinct, suggesting that MyD88 deficiency may disrupt signaling from TLRs other than TLR-2 and TLR-4 or disrupt signaling from MyD88-dependent cytokine receptors such as the IL-1 or IL-18 receptors. In both experiments, the *Trem1* transcript level, which is an indicator of macrophage activation, was similar to the wild type group, suggesting that macrophage activation was not different. More strikingly was the observation that both *Col1a1* and *Acta2* gene transcripts were reduced by approximately 50% in mice following kidney U-IRI and this was similar between MyD88 and TLR-2/TLR-4 deficiency (**Fig. 4B,E**). These gene transcripts are restricted to the pericyte/fibroblast/myofibroblast lineage, suggesting that deficiency of MyD88 and TLR-2/TLR-4 was impacting mesenchyme cell activation in the U-IRI model predominantly. Furthermore, the epithelial injury marker Kim-1 was elevated similarly in MyD88 or TLR-2/TLR-4 deficiency, suggesting that epithelial injury was not different between the mutant and wild type mice and that these activating receptors may not be critical in epithelial cells (**Fig. 4B,E**). In addition, the extent of αSMA+ myofibroblasts and the extent of macrophage recruitment to the kidneys was reduced when MyD88 or TLR-2/TLR-4 were deleted (**Fig. 4C,F**). Although deficiency of TLR-2, TLR-4 and MyD88 all had anti-inflammatory and anti-fibrotic effects in the U-IRI model, the UUO model was completely insensitive to deletion of these genes, indicating that the factors stimulating activation and injury in the UUO model did not involve the TLR-2, TLR-4 or MyD88 pathways (**Fig. S6**).

### The TLR-2/TLR-4/MyD88 Pathway is Dispensable in Macrophage Activation in Kidney Fibrosis, but Important in Mesenchymal Cell Activation

**Figure 5:**
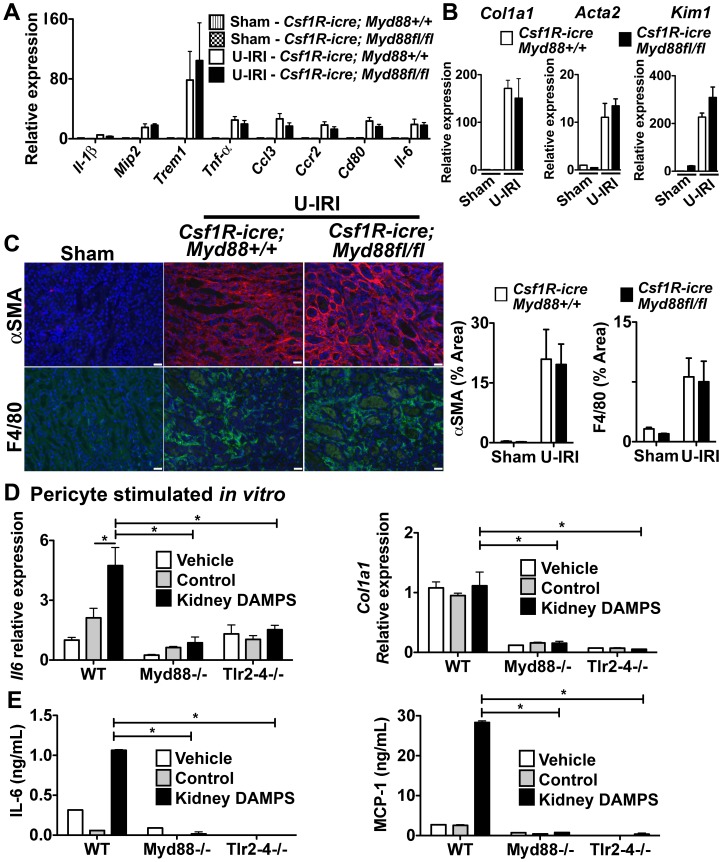
The TLR-2/TLR-4/MyD88 pathway is dispensable in macrophage activation in kidney fibrosis, but important in mesenchyme cell activation. (**A–C**) *Csf1R-icre; Myd88^fl/fl^* mice or respective controls were subjected to U-IRI and kidney harvested for tissue analysis 5 days later. Q-PCR (A) for different inflammatory transcripts, (**B**) pro-fibrotic transcripts, collagen1a1 (*col1a1*) and alpha smooth muscle actin (*Acta2*), and the tubule injury marker, kidney injury molecule-1 (*Kim-1*) from whole kidney day 5 after U-IRI. (**C**) Representative fluorescent images (left) and quantitative graphs (right) showing+αSMA (red) cells and+F4/80 cells (green). (**D–E**) Primary pericytes were isolated from *Myd88^−/−^* and *Tlr2–4^−/−^* mice and stimulated *in vitro* for 8h with kidney DAMPs. (**D**) Graph showing *Il-6* and *Col1a1* transcript expression by Q-PCR. (**E**) Graph showing IL-6 and MCP-1 concentration in supernatant by ELISA. (*P<0.05, n = 5–7/group, 3 independent experiments; ns, p is not significant; Bar = 50 µm; Q-PCR results were normalized to wild type control).

To test the importance of these activating receptors in myeloid cells further, we generated mice lacking MyD88 somatically in myeloid lineage cells (macrophages, dendritic cells and neutrophils) *Csf1R-iCre; Myd88^fl/fl^* [29]. To validate the deletion of MyD88 in myeloid lineage cells, we generated BMDMφs from these mice and *Csf1R-iCre; Myd88^+/+^* littermates. As expected the WT mice expressed high levels of MyD88, which was further induced by LPS (**Fig. S7A**), but *Csf1R-iCre; Myd88^fl/fl^* showed no detectable MyD88 protein. Similarly, BMDMφs from *Csf1R-iCre; Myd88^fl/fl^* mice were completely insensitive to LPS (**Fig. S7B**), a result phenocopied using BMDMφs from mice with germline MyD88 deficiency. These results indicate that MyD88 is completely deleted in myeloid lineage cells in the *Csf1R-iCre; Myd88^fl/fl^* mice. Unexpectedly, deletion of MyD88 only in myeloid cells resulted in no difference in pro-inflammatory cytokines or chemokines in the U-IRI kidney injury model (**Fig. 5A**). In addition, whereas the MyD88 deficient mice showed protection from myofibroblast activation **(** **Fig. 4E** **)**, *Csf1R-iCre; Myd88^fl/fl^* mice showed no evidence of a reduction in myofibroblast activation **(** **Fig. 5B** **)** and the extent of αSMA+ myofibroblasts **(** **Fig. 5C** **)**. Although the *Csf1R-iCre; Myd88^fl/fl^* mice were an F2 generation of FVB crossed with C57bl/6 mouse strain, the extent of disease observed in the *Csf1R-iCre; Myd88^+/+^* was identical to C57bl/6 wild type (Fig. 4), therefore these findings suggest that in the U-IRI model, these pathways are dispensable for macrophage activation.

Because TLR-2, TLR-4 and Myd88 germline deficiency had marked affects on myofibroblast activation and fibrogenesis, we hypothesized that these receptors may be important mesenchymal cell (myofibroblast progenitor) activation. We therefore generated primary cultures of kidney pericytes (myofibroblast progenitors) and stimulated them with kidney DAMPs. Pericytes robustly activated *Il-6* and MCP-1 (**Fig. 5D–E**) but not *Il-1β* in response to DAMPs (Data not shown). Our studies predicted that MyD88, TLR-2 and TLR-4 may play a role in DAMP- mediated activation in pericytes. To test these we cultured MyD88, and TLR-2/TLR-4 deficient pericytes. Pericytes deficient in MyD88 or TLR-2/TLR-4 were not activated when stimulated with kidney DAMPs (**Fig. 5D–E**). Pericytes in culture express high-baseline levels of *Col1a1* transcript, and DAMPs did not further increase *Col1a1* production. However, pericytes deficient in MyD88 and TLR-2/TLR-4 expressed very low baseline levels of *Col1a1* transcript when compared to wild type pericytes, and stimulation with kidney DAMPs did not induce *Col1a1* (**Fig. 5D**), indicating that TLR-2/TLR-4 and MyD88 pathways are important for mesenchyme cell activation.

## Discussion

These studies show that although TREM-1 is highly upregulated in two models of chronic kidney disease in mice, and can regulate TLR sensitivity to LPS, it plays no role in sterile activation of monocytes/macrophages that are recruited to the injured kidney. These studies also show that, although the TLR-2, TLR-4 and MyD88 signaling pathways may play a small role in activation of macrophages by kidney DAMPs *in vitro*, they are largely dispensable *in vivo*. However, MyD88-dependent TLR-2, TLR-4 signaling appears to be critical in activation of mesenchymal cells of the kidney.

TREM genes were highly upregulated in the kidney in Ly6C^+^ macrophages in response to injury. Several studies report a similar response in sterile inflammation in other organs, and report that TREM-1 blockade either by TREM1-Fc or by a peptide, attenuates the disease process [23]–[26]. Our studies in the kidney do not support a functional role for TREM-1 despite the similarities between the studies. This may reflect differences in DAMPs released in different organs, or the differing roles of macrophages in each tissue. The fact that the mouse TREM-3 gene lies adjacent to the TREM-1, and studies have shown that both receptors have redundant functions in the mouse [43], [44], suggests that TREM-3 could be compensating for TREM-1 in our model. However, like TREM-1, TREM-3 is reported to signal through DAP12 to function as an amplifier of inflammation. Our findings indicate that *Dap12^−/−^* mice (Table **S1**) and Trem-2^−/−^ mice (not shown) had similar disease severity compared to strain-matched controls, suggesting that these pathways are dispensable in these models of kidney injury.

The studies presented here show a novel method for studying kidney DAMPs. The preparation method although crude, robustly and reliably activates leukocytes and can be used to dissect individual DAMP factors. The fact that DAMPs are heat sensitive, but not sensitive to trypsin and pronase, indicates that they may be non-proteinaceous factors, including nucleic acids, lipids or products of extracellular matrix. However, they also could be protein complexes resistant to proteolytic digestion. Consistent with this a known DAMP, HMGB1 was readily identified in the preparation. Further studies beyond the scope of the current studies should identify these DAMPs.

Because TLRs have been implicated as effectors in kidney diseases [45], [46] and TREM-1 has been previously linked to TLR signaling, we investigated TLR-2 and TLR-4 as putative receptors for kidney DAMPs and macrophage activation. The studies presented here show a significant role for TLR-2, TLR-4 and MyD88 *in vivo*, particularly in the U-IRI model of chronic inflammation, but indicate they are not important in macrophage activation, even though macrophages during chronic kidney injury contribute to disease progression and fibrogenesis [2] [3]. Both the *in vitro* studies and *in vivo* studies implicate other signaling pathways that are more important in macrophage activation in kidney disease. Strikingly, we also identified C-type lectin (Clec) 4d and Clec4e as kidney macrophage PRRs in the transcriptional profiling experiments. Clec4e, also known as MINCLE, was reported to be a critical PRR in macrophages responding to the mitochrondrial spliceosomal protein SAP130, and signals through FcRγ [47]. We previously published that FcRγ deficient mice exhibit a significant reduction in injury and fibrosis in these models of kidney injury. However the interacting receptors involved in this activation were not determined [35]. Since these current studies have ruled out a role for TREM-1 in macrophage activation in the kidney future studies should determine whether the Clec receptors play dominant roles via FcRγ in kidney macrophage activation.

Although TLR signaling has been implicated in kidney diseases in other studies [45], the role of these receptors in different cell compartments in the kidney has been controversial and has been thought to depend on the model of injury. Using a bone marrow chimeric approach, it has been suggested that TLRs may play a more important role in parenchymal cells rather than myeloid cells during acute kidney injury [48], [49]. Our findings would support that. However our studies do not indicate that epithelial cell injury is reduced when MyD88 or TLR-2 and TLR-4 are absent, implicating other kidney cells. Until recently, the existence of the mesenchymal cells in the kidney, known as pericytes and resident fibroblasts, has been underappreciated but with new genetic tools their roles in fibrogenesis and innate immunity as well as vascular biology have recently become established [33], [37], [50]. Pericytes are embedded in the peritubular capillaries of the kidney and, like dendritic cells, form a barrier at the vascular interface [51]–[53]. Using a microarray approach, we have recently published that during a progressive kidney disease, genes involved in the immune response were highly upregulated in the pericyte to myofibroblast transition in the kidney [37]. Although surprising, our studies suggest that pericytes may form an important early immune defense to injury by release of pro-inflammatory cytokines and chemokines and that this response is highly dependent on TLR-2, TLR-4 and MyD88. Further studies are required.

We conclude that TREM-1 and TLR-2, TLR-4 and MyD88 are dispensable in macrophage activation in sterile kidney injury but that these pathways are important in activation of kidney pericytes.

## Supporting Information

Figure S1 **Ly6C Macrophage subpopulations purified from UUO kidney.** (**A**) Representative plots of total kidney cells from single cell preparation 5 days after UUO were selected for viability and singularity by initial forward and side scatter gates. (**B**) Ly6G+ and NK1.1+ cells were negative gated to exclude neutrophils and NK cells. The different macrophage subpopulation were sorted by gating populations of CD11b+ cells with three levels of Ly6C expression: Ly6C^high^, Ly6C^int^, and Ly6^low^.
(TIF)

Figure S2 **Transcriptional analysis of activated macrophages in sterile kidney injury.** Clustered profiles of 63 differentially expressed genes between Ly6C^+^ (Ly6C^high^ and Ly6C^int^, n = 3/group) and Ly6C^low^ (n = 2) macrophages depicted using a heatmap. Note the progressive decline in *Trem1* expression levels across Ly6C^high^, Ly6C^int^, and Ly6C^low^ sub-populations. Gene Ontology relational representation of highly enriched functional modules corresponding to differentially expressed genes between Ly6C^+^ and Ly6C^low^ macrophages. Prominent processes include immune response, migration, chemotaxis, and cytokine binding and activity.
(TIF)

Figure S3 **Temperature sensitive kidney DAMPs activate macrophages ex vivo.** (**A**) Q-PCR for *Il-1β* in BMDMφ primed with IFNγ (0, 250 or 500 U/ml) for 8 hours, washed, and further stimulated with kidney DAMPs for 12 hours. (**B**) Coomassie blue stained SDS PAGE of crude preparation of soluble extracellular factors from normal (control) and disease kidney (kidney DAMPs). (**C**) Western blotting showing HMGB1 expression in soluble extracellular factors from control and kidney DAMPs. (**D**) Q-PCRs from BMDMφs treated with DAMPs for 16 h showing the effect of temperature changes on kidney DAMP activity. (**E**) Q-PCR showing the effect of kidney DAMPs digestion for 16 h with Trypsin (1∶20 w/w ratio) or Pronase (1∶50 w/w ratio) prior application to BMDMφ for 16 h. (*P<0.05, n = 5–7/group, 3 independent experiments; ns, p is not significant).
(TIF)

Figure S4 **TREM-1 pathway is important for BMDMφ activation by LPS** ***in vitro*** **, but dispensable for activation by kidney DAMPs.** (**A**) Schema of TREM1-Fc fusion protein and Western blot of purified TREM1-Fc, detected by anti-TREM-1 antibodies. **(B–C)** Q-PCR for *Il-1β* in BMDMφs stimulated with LPS and treated with (**B**) TREM1-Fc or (**C**) anti-TREM-1 antibodies. (**D**) Q-PCR for *Il-1β* in BMDMφ pre-incubated with kidney DAMPs for 8 h to induce TREM-1 expression, followed by kidney DAMPs in the presence of anti-TREM-1 antibodies or TREM1-Fc for 16 h further. (**E**) Q-PCR showing BMDMφ response to DAMPs for 16 h that were pre-adsorbed by hIgG or TREM1-Fc coated protein-A beads. **(F–G)** Colorimetric assay reporting Lacz activity in BWZ-Lacz reporter cells expressing TREM1-DAP12 chimera protein stimulated with kidney DAMPs for 16 h in wells (**F**) pre-coated with kidney DAMPs or (**G**) in suspension (anti-TREM-1 antibodies are positive control). (n = 3–5/group, 3 independent experiments; *P<0.05).
(TIF)

Figure S5 **Treatment with soluble TREM1-Fc does not prevent macrophage activation, injury and fibrosis in UUO model of sterile kidney injury.** Mice were subjected to unilateral ureter obstruction (UUO) and treated daily with 40 µg/mouse of TREM1-Fc or hIgG, as control. (**A**) Q-PCR for different inflammatory transcripts (left) or pro-fibrotic transcripts, Collagen1a1 *(Col1a1)* and alpha smooth muscle actin (*Acta2*), from whole kidney day 5 after UUO. (**B**) Representative images (left) and quantitative graphs (right) showing+F4/80 cells (green),+αSMA (red) or collagen deposition (Sirius Red staining) day 5 after UUO. (*P<0.05, n = 5–7/group, 3 independent experiments; Bar marker = 50 µm; Q-PCR data were normalized to sham+higG control).
(TIF)

Figure S6 **The TLR2/4/MyD88 pathway is dispensable in the UUO model of sterile kidney injury. (A,C,E)** Q-PCR for different inflammatory molecules, pro-fibrotic transcripts, collagen1a1 (*col1a1*) and alpha smooth muscle actin (*Acta2*), and the tubule injury marker, kidney injury molecule-1 (*Kim-1*) from whole kidney day 5 after UUO in (**A**) *Myd88^−/−^,* (**C**) *Tlr2–4^−/−^*, and mice lacking MyD88 only in myeloid cells lineage, (**E**) *Csf1R-icre; MyD88^fl/fl^*. (**B,D,F**) Graphs showing quantification of fluorescent images for+αSMA cells and+F4/80 cells. (*P<0.05, n = 5–7/group; Q-PCR data were normalized to wild type sham).
(TIF)

Figure S7 **Validation of MyD88 conditional ablation in myeloid cells expressing Csf1R.** *Csf1R-iCre* mice were crossed with *Myd88^fl/fl^* to generate *Csf1R-icre; Myd88^fl/fl^* mice, which selectively ablates MyD88 expression in myeloid cells expressing Csf1R. (**A**) Western blot showing basal or LPS-induced MyD88 expression of BMDMφ isolated from *Csf1R-icre; Myd88^+/+^* or *Csf1R-icre; MyD88^fl/fl^*. (**B**) Q-PCR for *Il-1β* expression of BMDMφ from *Csf1R-icre; Myd88^+/+^*, *Csf1R-icre; MyD88^fl/fl^*, *Myd88^+/+^* and *Myd88^−/−^* mice stimulated with LPS for 16h. (*P<0.05, n = 3–5/group; Q-PCR data were normalized to wild type control).
(TIF)

Table S1 **Quantitative PCR from kidney tissue day 5 after U-IRI injury.**
(DOCX)

Methods S1. (DOCX)
