# Supplementary figures and images for: TLR-2/TLR-4 TREM-1 Signaling Pathway Is Dispensable in Inflammatory Myeloid Cells during Sterile Kidney Injury
Source: PLoS One. 2013 Jul 3;8(7):e68640. doi: 10.1371/journal.pone.0068640 (PMC3700949; doi:10.1371/journal.pone.0068640)

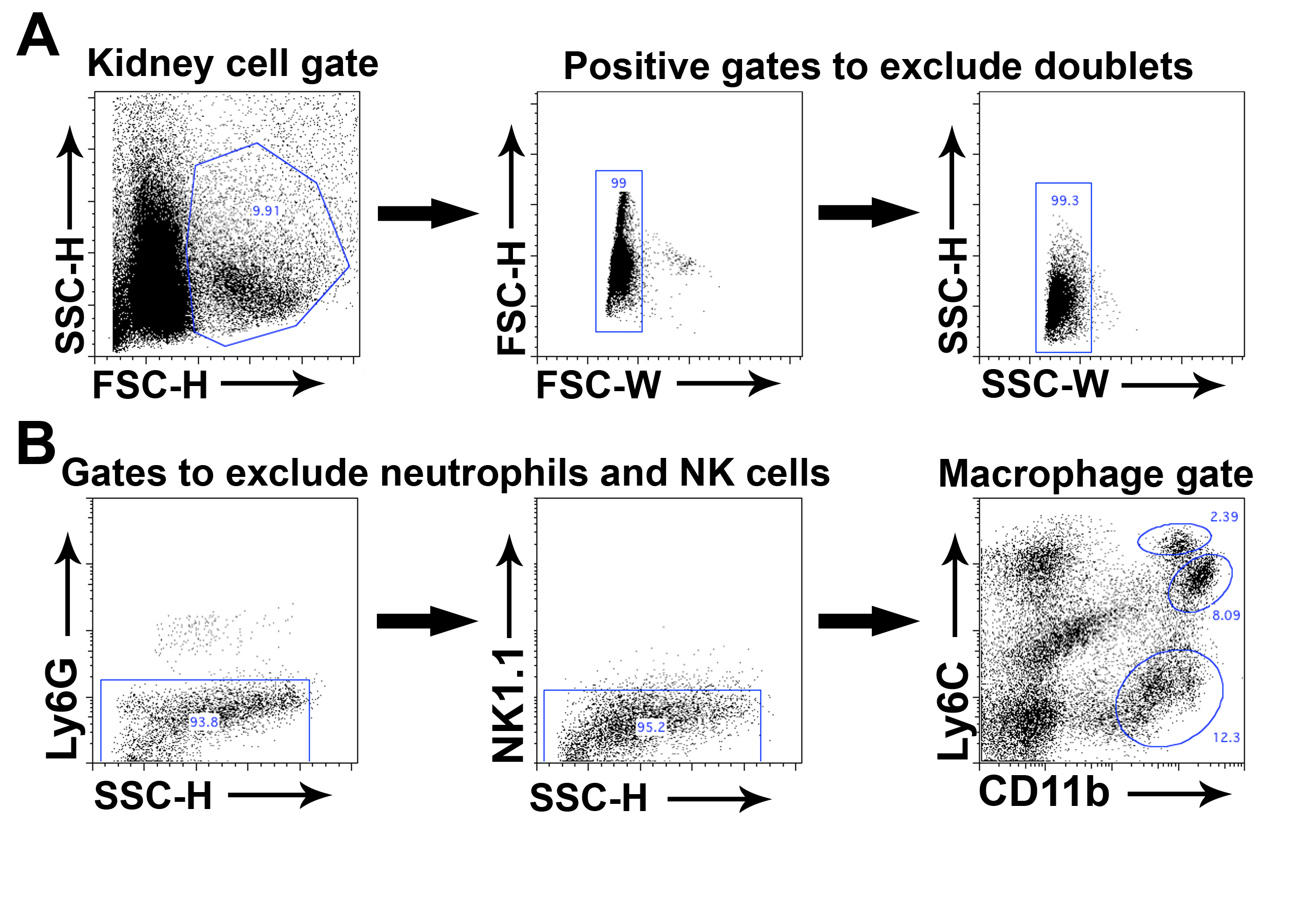

Supplement: Figure S1 — Ly6C Macrophage subpopulations purified from UUO kidney. (A) Representative plots of total kidney cells from single cell preparation 5 days after UUO were selected for viability and singularity by initial forward and side scatter gates. (B) Ly6G+ and NK1.1+ cells were negative gated to exclude neutrophils and NK cells. The different macrophage subpopulation were sorted by gating populations of CD11b+ cells with three levels of Ly6C expression: Ly6Chigh, Ly6Cint, and Ly6low. (TIF) [file pone.0068640.s001.tif]

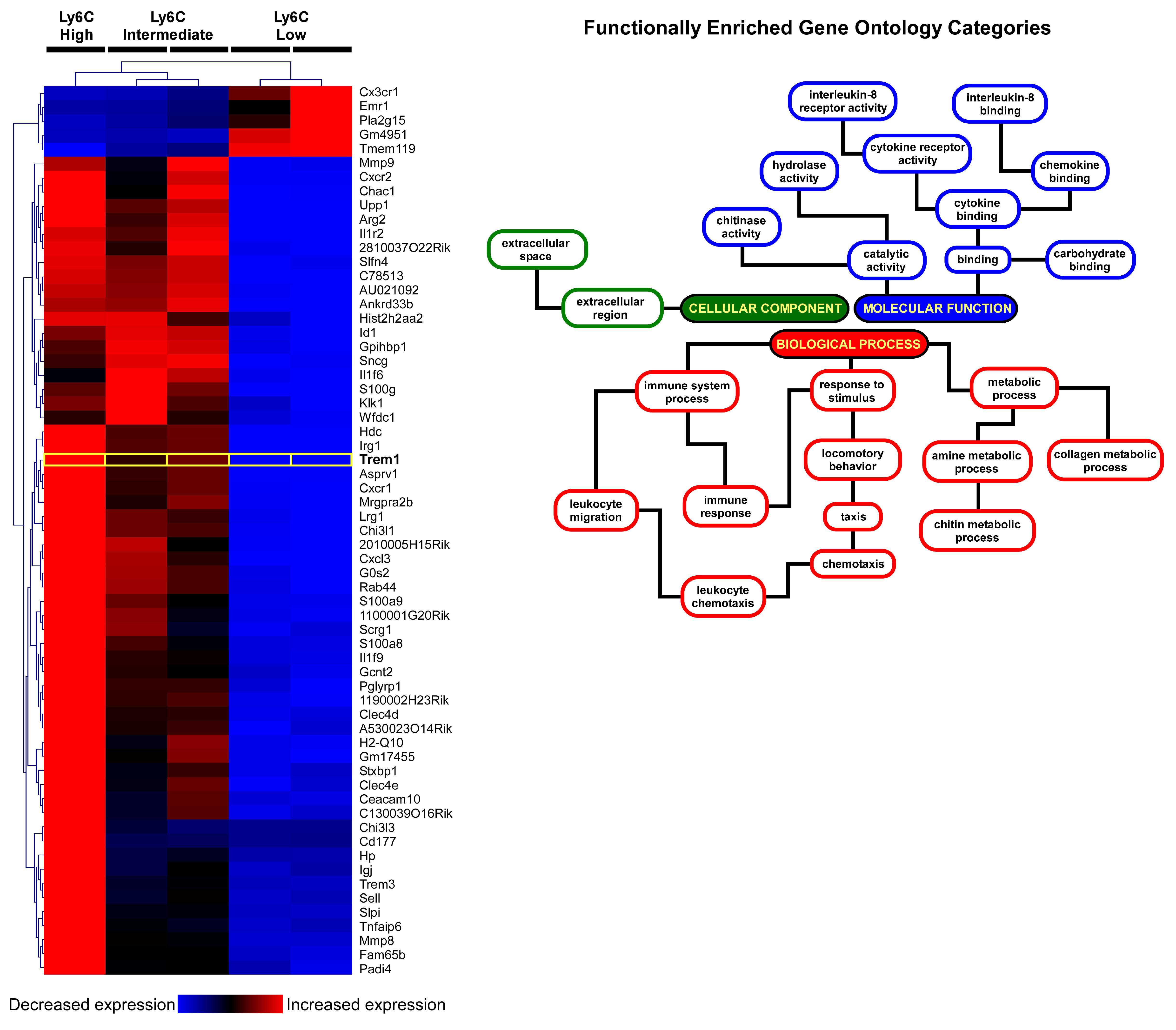

Supplement: Figure S2 — Transcriptional analysis of activated macrophages in sterile kidney injury. Clustered profiles of 63 differentially expressed genes between Ly6C+ (Ly6Chigh and Ly6Cint, n = 3/group) and Ly6Clow (n = 2) macrophages depicted using a heatmap. Note the progressive decline in Trem1 expression levels across Ly6Chigh, Ly6Cint, and Ly6Clow sub-populations. Gene Ontology relational representation of highly enriched functional modules corresponding to differentially expressed genes between Ly6C+ and Ly6Clow macrophages. Prominent processes include immune response, migration, chemotaxis, and cytokine binding and activity. (TIF) [file pone.0068640.s002.tif]

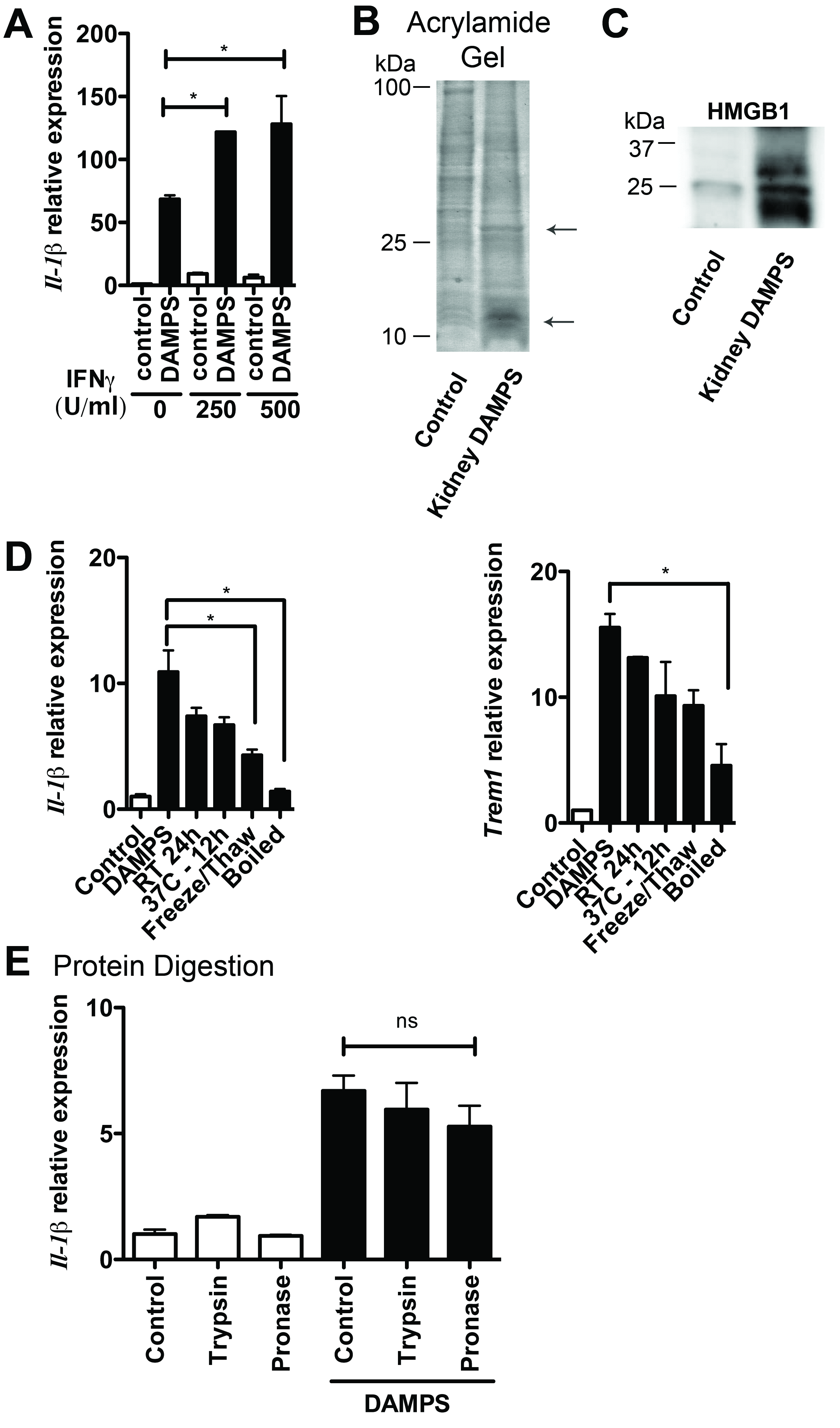

Supplement: Figure S3 — Temperature sensitive kidney DAMPs activate macrophages ex vivo. (A) Q-PCR for Il-1β in BMDMφ primed with IFNγ (0, 250 or 500 U/ml) for 8 hours, washed, and further stimulated with kidney DAMPs for 12 hours. (B) Coomassie blue stained SDS PAGE of crude preparation of soluble extracellular factors from normal (control) and disease kidney (kidney DAMPs). (C) Western blotting showing HMGB1 expression in soluble extracellular factors from control and kidney DAMPs. (D) Q-PCRs from BMDMφs treated with DAMPs for 16 h showing the effect of temperature changes on kidney DAMP activity. (E) Q-PCR showing the effect of kidney DAMPs digestion for 16 h with Trypsin (1∶20 w/w ratio) or Pronase (1∶50 w/w ratio) prior application to BMDMφ for 16 h. (*P<0.05, n = 5–7/group, 3 independent experiments; ns, p is not significant). (TIF) [file pone.0068640.s003.tif]

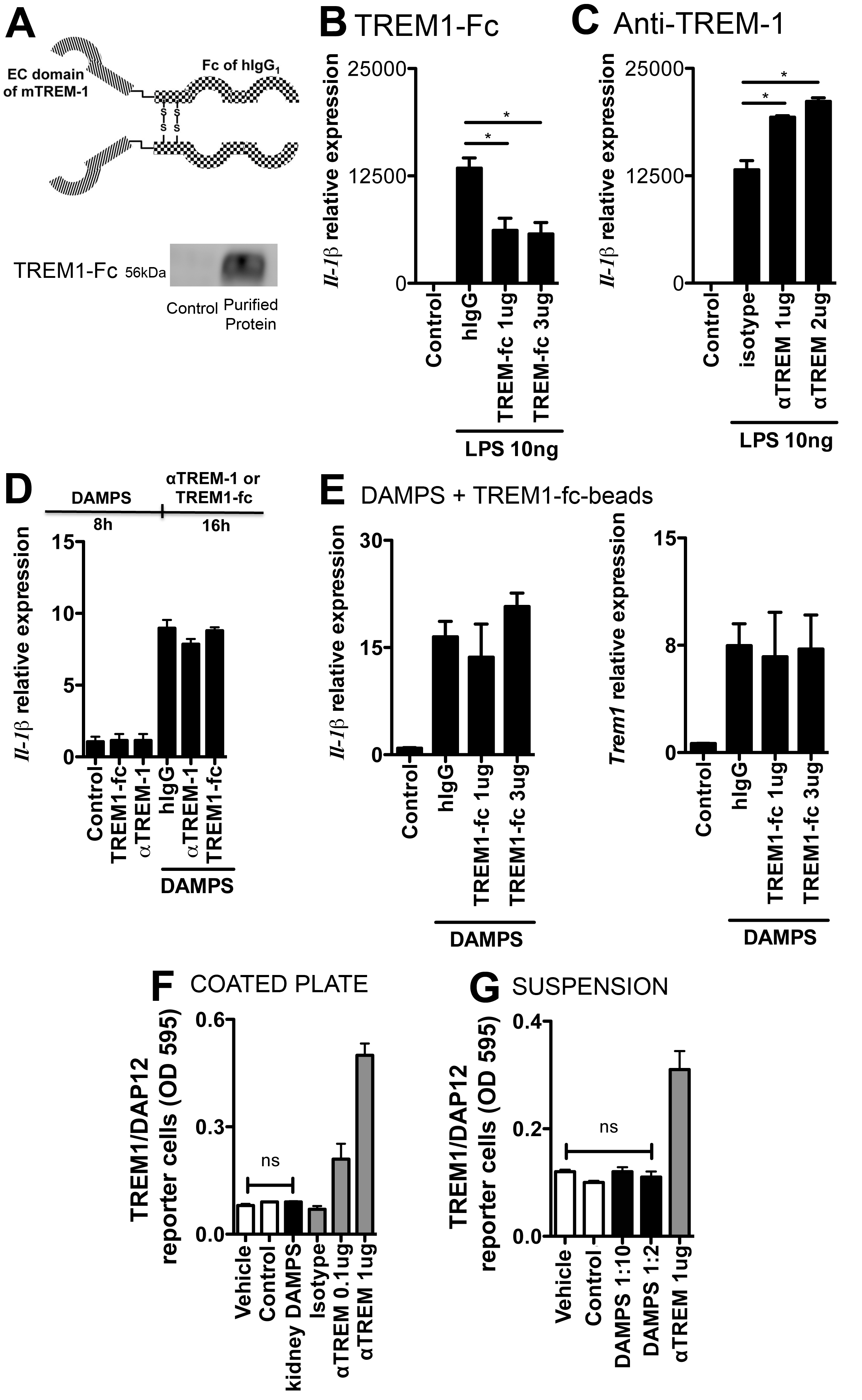

Supplement: Figure S4 — TREM-1 pathway is important for BMDMφ activation by LPS in vitro , but dispensable for activation by kidney DAMPs. (A) Schema of TREM1-Fc fusion protein and Western blot of purified TREM1-Fc, detected by anti-TREM-1 antibodies. (B–C) Q-PCR for Il-1β in BMDMφs stimulated with LPS and treated with (B) TREM1-Fc or (C) anti-TREM-1 antibodies. (D) Q-PCR for Il-1β in BMDMφ pre-incubated with kidney DAMPs for 8 h to induce TREM-1 expression, followed by kidney DAMPs in the presence of anti-TREM-1 antibodies or TREM1-Fc for 16 h further. (E) Q-PCR showing BMDMφ response to DAMPs for 16 h that were pre-adsorbed by hIgG or TREM1-Fc coated protein-A beads. (F–G) Colorimetric assay reporting Lacz activity in BWZ-Lacz reporter cells expressing TREM1-DAP12 chimera protein stimulated with kidney DAMPs for 16 h in wells (F) pre-coated with kidney DAMPs or (G) in suspension (anti-TREM-1 antibodies are positive control). (n = 3–5/group, 3 independent experiments; *P<0.05). (TIF) [file pone.0068640.s004.tif]

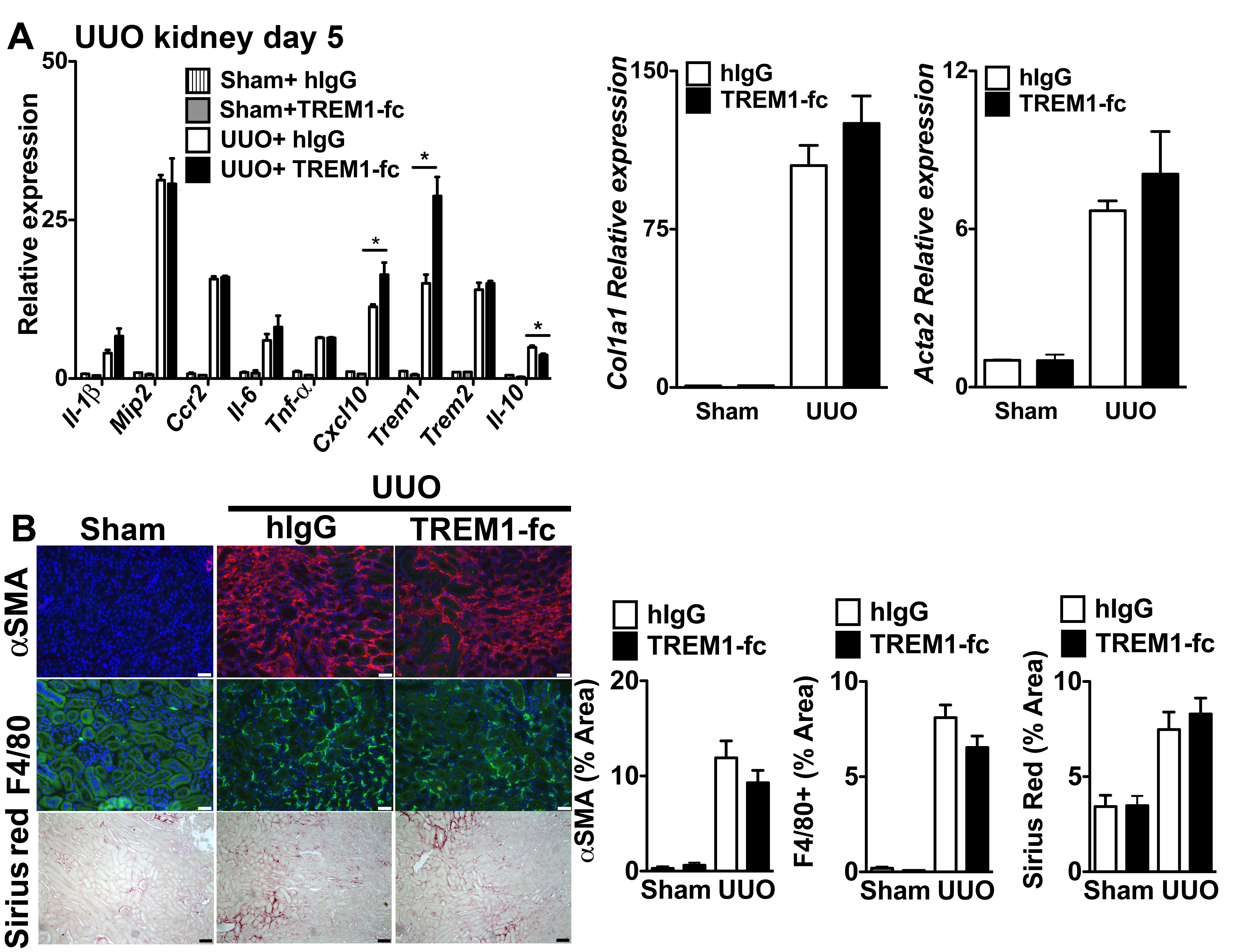

Supplement: Figure S5 — Treatment with soluble TREM1-Fc does not prevent macrophage activation, injury and fibrosis in UUO model of sterile kidney injury. Mice were subjected to unilateral ureter obstruction (UUO) and treated daily with 40 µg/mouse of TREM1-Fc or hIgG, as control. (A) Q-PCR for different inflammatory transcripts (left) or pro-fibrotic transcripts, Collagen1a1 (Col1a1) and alpha smooth muscle actin (Acta2), from whole kidney day 5 after UUO. (B) Representative images (left) and quantitative graphs (right) showing+F4/80 cells (green),+αSMA (red) or collagen deposition (Sirius Red staining) day 5 after UUO. (*P<0.05, n = 5–7/group, 3 independent experiments; Bar marker = 50 µm; Q-PCR data were normalized to sham+higG control). (TIF) [file pone.0068640.s005.tif]

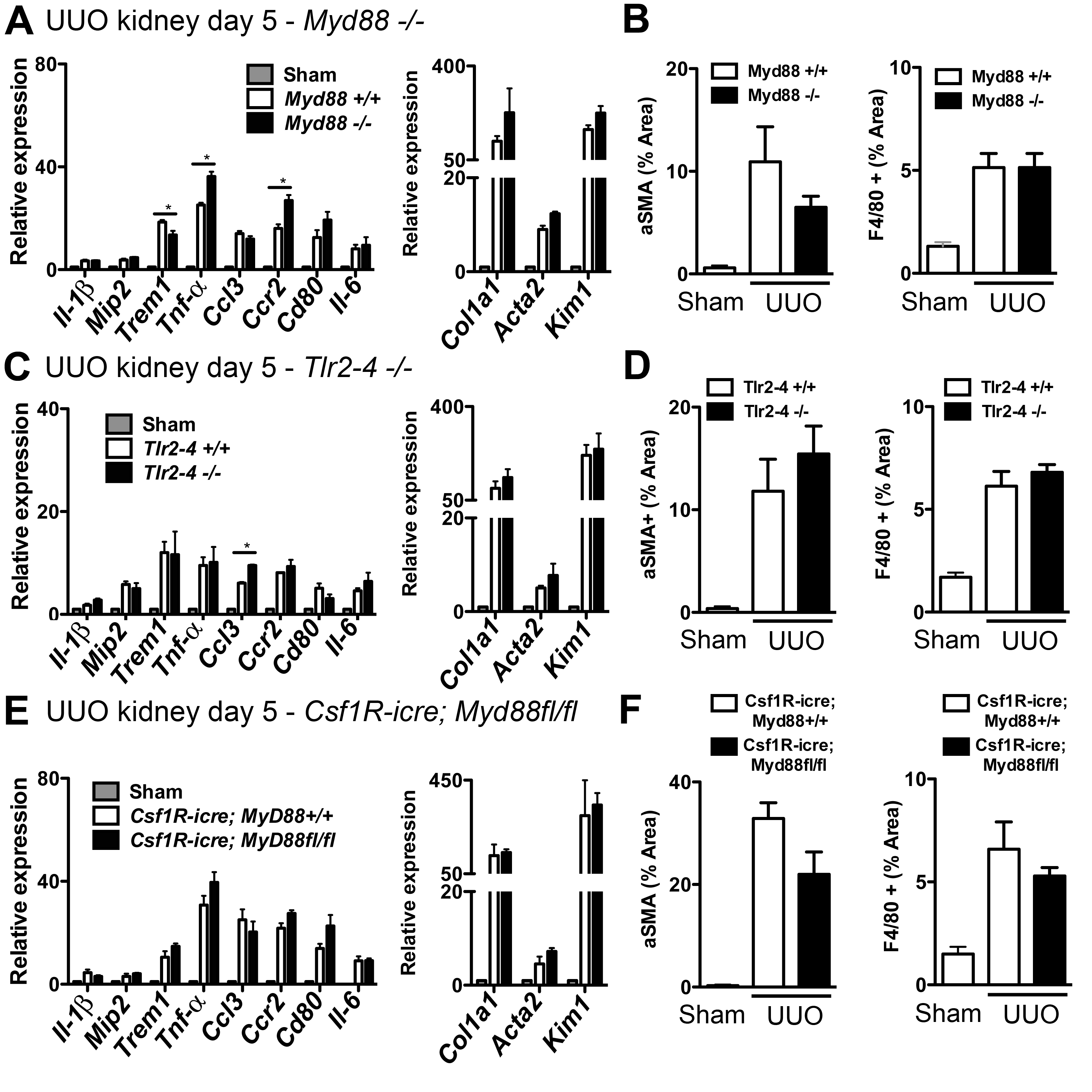

Supplement: Figure S6 — The TLR2/4/MyD88 pathway is dispensable in the UUO model of sterile kidney injury. (A,C,E) Q-PCR for different inflammatory molecules, pro-fibrotic transcripts, collagen1a1 (col1a1) and alpha smooth muscle actin (Acta2), and the tubule injury marker, kidney injury molecule-1 (Kim-1) from whole kidney day 5 after UUO in (A) Myd88−/−, (C) Tlr2–4−/−, and mice lacking MyD88 only in myeloid cells lineage, (E) Csf1R-icre; MyD88fl/fl. (B,D,F) Graphs showing quantification of fluorescent images for+αSMA cells and+F4/80 cells. (*P<0.05, n = 5–7/group; Q-PCR data were normalized to wild type sham). (TIF) [file pone.0068640.s006.tif]

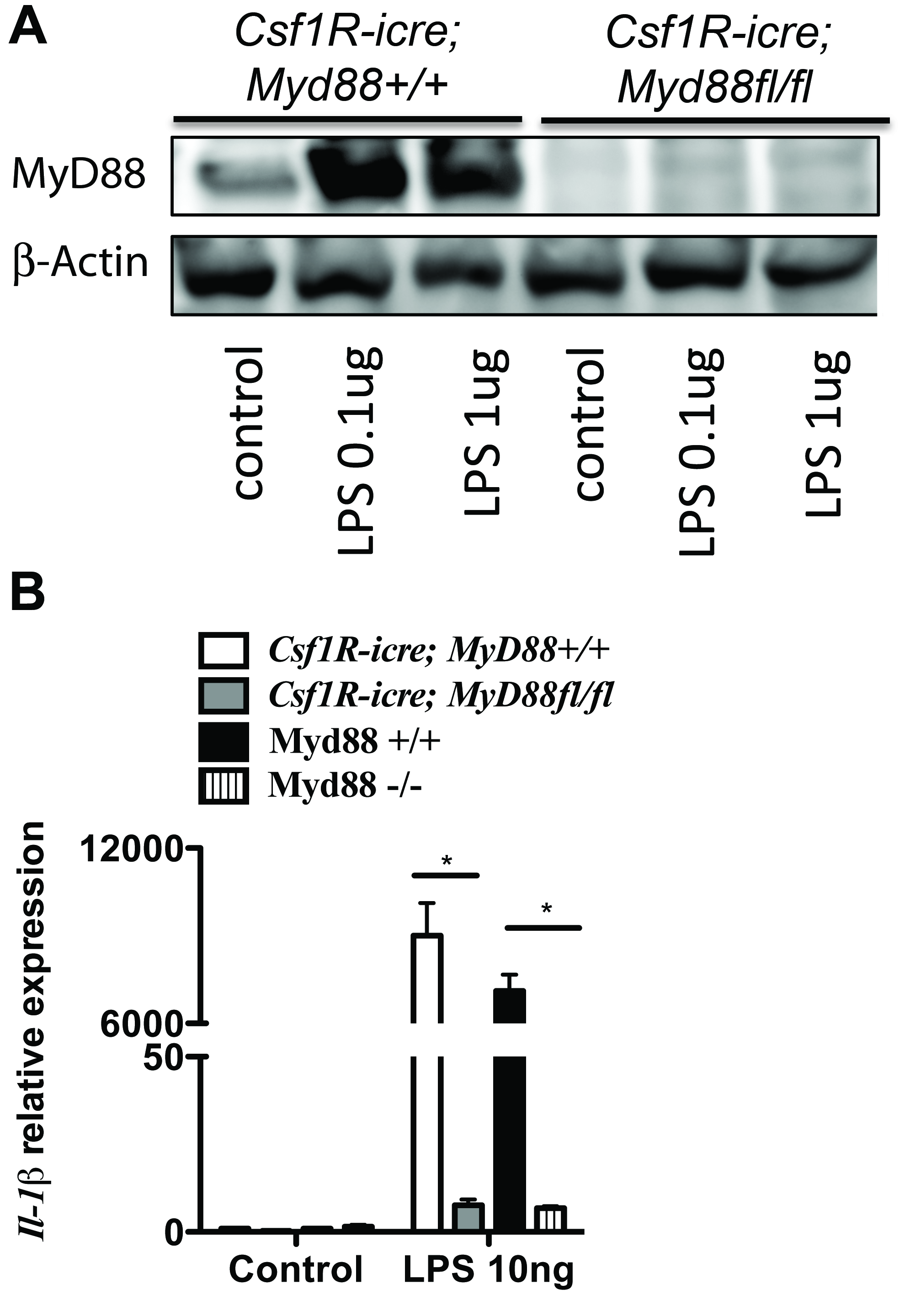

Supplement: Figure S7 — Validation of MyD88 conditional ablation in myeloid cells expressing Csf1R. Csf1R-iCre mice were crossed with Myd88fl/fl to generate Csf1R-icre; Myd88fl/fl mice, which selectively ablates MyD88 expression in myeloid cells expressing Csf1R. (A) Western blot showing basal or LPS-induced MyD88 expression of BMDMφ isolated from Csf1R-icre; Myd88+/+ or Csf1R-icre; MyD88fl/fl. (B) Q-PCR for Il-1β expression of BMDMφ from Csf1R-icre; Myd88+/+, Csf1R-icre; MyD88fl/fl, Myd88+/+ and Myd88−/− mice stimulated with LPS for 16h. (*P<0.05, n = 3–5/group; Q-PCR data were normalized to wild type control). (TIF) [file pone.0068640.s007.tif]
