# Supplementary material for: TLR-2/TLR-4 TREM-1 Signaling Pathway Is Dispensable in Inflammatory Myeloid Cells during Sterile Kidney Injury
Source: PLoS One. 2013 Jul 3;8(7):e68640. doi: 10.1371/journal.pone.0068640 (PMC3700949; doi:10.1371/journal.pone.0068640)
Supplement: Table S1 — Quantitative PCR from kidney tissue day 5 after U-IRI injury. (DOCX) [file pone.0068640.s008.docx]

**SI Table**

**Table S1. Quantitative PCR from kidney tissue day 5 after U-IRI injury.**

|  | **SHAM** | | **IRI** | |
| --- | --- | --- | --- | --- |
|  | **WT** | ***Dap12-/-*** | **WT** | ***Dap12-/-*** |
| Mip2 | 1±0.3 | 0.9±0.4 | 3.3±0.3 | 3.3±0.4 |
| Trem1 | 1±0.4 | 0.6 ±0.1 | 8.5±0.7 | 10.6±1.9 |
| Il-1β | 1±0.2 | 1.3±0.6 | 4.7±0.7 | 5±1 |
| Ccr2 | 1±0.1 | 0.9±0.1 | 2.5±0.1 | 2.5±0.2 |
| Il-10 | 1±0.1 | 1.2±0.47 | 4.6±0.9 | 4.9±0.2 |
| Tnf-α | 1±0.1 | 1.3±0.47 | 8.2±0.5 | 10.4±1.7 |
