# Supplementary material for: TLR-2/TLR-4 TREM-1 Signaling Pathway Is Dispensable in Inflammatory Myeloid Cells during Sterile Kidney Injury
Source: PLoS One. 2013 Jul 3;8(7):e68640. doi: 10.1371/journal.pone.0068640 (PMC3700949; doi:10.1371/journal.pone.0068640)
Supplement: Methods S1. — (DOCX) [file pone.0068640.s009.docx]

**SUPPORTING INFORMATION**

**SI Methods**

**Purification of kidney Macrophage subpopulations for microarray analysis**

Kidneys from day 5 UUO were collected and single cell preparation was performed as previously described [1],[2]. Single cells were resuspended on ice in 2ml FACS buffer (PBS, 1% BSA, 2mM EDTA (pH 7.4)) and antibodies against the following cell surface markers applied under sterile conditions CD11b (APC, 1:500 E-bioscience), NK1.1 (PE-Cy5.5 1:300 E-Bioscience), Ly6G (PE BD Pharmingen), Ly6C (FITC BD Pharmingen). The total single cell preparation was analyzed directly by FACSAria cell sorter. After cells were selected by FSC and SSC and doublets were gated out, negative gates were removed Ly6G and NK1.1 positive cells. Positive gates were selected around populations of CD11b+ cells with three levels of Ly6C expression: Ly6C^high^, Ly6C^int^, and Ly6^low^. Cells were sorted at 4°C directly into 600µl RLT buffer (Qiagen). Cells in buffer were vortexed for 30s and stored at -80°C, prior to RNA purification using RNA Easy (Qiagen). The quality of RNA was tested by Agilent Bioanalyzer and high quality samples underwent RNA amplification by *in vitro* transcription method at Harvard University Core Microarray Biotechnology Center. cRNA samples were hybridized to Codelink mouse whole genome microarrays using the manufacturer’s protocols (Applied Microarrays, Tempe, AZ) and scanned using Axon GenePix scanner (Molecular Devices, Sunnyvale, CA). Image processing, background subtraction, and median-based normalization of probeset intensities were performed using Codelink’s Expression Analysis Software (Applied Microarrays, Tempe, AZ).

**Microarray data analysis**

Differential gene expression between Ly6C^+^ (Ly6C^high^ and Ly6C^int^, n = 3) and Ly6C^low^ (n = 2) macrophages was determined using a Bayesian implementation of the parametric *t*-test developed specifically for low replication microarray experiments [3],[4]. Multiple comparisons correction was performed using the *Q*-value method [5], with a statistical significance cutoff *Q*-value < 0.01. Differentially expressed genes underwent two-dimensional hierarchical clustering based on Pearson’s correlation [6] and functional analysis using the web-based program WebGestalt [7]. Enriched functional modules (false discovery rate < 0.01) were depicted based on Gene Ontology’s hierarchical structure [8].

**SDS PAGE**

To verify presence of proteins in the DAMPs preparation, the crude preparation of DAMPs from a normal kidney (control) or disease kidney was concentrated 10-fold using trichloroacetic acid. 15 μL of concentrated proteins added to 4-20% acrylamide gel (Bio-rad). After electrophoresis, the gel was stained for 1 hour with 0.25% Coomassie brilliant blue R-250 (Amresco), and destained overnight with destaining solution (45% ddH_2_O, 45% methanol, 10% acetic acid).

**BWZ TREM-1/DAP-12 reporter assay**

The BWZ mouse T cell lymphoma line containing the *Lacz* gene under the regulation of the NFAT promoter [9] was co-transfected with chimeric TREM-1/DAP-12 construct as previously described [10]. Cells were grown in RPMI 1640 with 10% FBS, 2mM l-glutamine, 25µM 2-ME, 100 U/ml penicillin and 100µg/ml streptomycin. Experiments were performed as described [10]-[12]. Briefly, 5x10^4^ BWZ.TREM1/DAP12 cells or control BWZ cells (parental BWZ) were seeded in wells of round bottom 96-well plates and stimulated in serum-free RPMI medium (final volume 100µl/well), with different dilutions of kidney DAMPS (1:1, 1:2 and 1:10), anti-TREM-1 monoclonal antibodies (0.1 or 1µg/well, R&D Systems), as a positive control, and isotype IgG_2a_, as a negative control. In other experiments, flat-bottom 96-well plates were coated with anti-TREM-1 antibodies (0.1 or 1μg/well), isotype control IgG_2a_, or DAMPs for 16h at 18°C or 4°C. After aspiration, BWZ.TREM1/DAP12 or parental cells were added in 100µl. Plates were incubated 16h, 37°C, washed 3X with PBS, then cells were lysed in 150µM chlorophenol red-B-D-galactopyranoside (Calbiochem), 9mM MgCl_2_, 0.125% Nonidet P-40 in PBS and incubated 8h at 37°C. β-galactosidase activity was measured by reading absorbance at 595nm.

**Bone Marrow Macrophage stimulation**

BMDMφ were stimulated with 10ng or 100ng/ml of LPS (E. coli O111:B4, Invivogen) or mouse IFNγ (Peprotech) for 8h at 250 or 500U/ml. In other experiments, kidney DAMPs were subjected to room temperature (RT) for 24h, 37°C for 12h, freeze (-80°C) and thaw (RT) X3, boiling for 5min, or protein digestion before adding to BMDMφ. For protein digestion, total protein concentration of crude DAMPs was measured by Bradford Assay (Bio-rad) and Trypsin (Promega) or Pronase (Sigma) was added to DAMPs in 1:20 or 1:50 ratio (weight/weight), respectively, and digested 16h, 37°C. In some experiments, TREM1-Fc (1 or 3μg) was conjugated with sepharose Protein-A beads (Invitrogen) (10μl), incubated with kidney DAMPs 16h, 4°C, centrifuged and the supernatant was used to stimulate BMDMφ.

**SI References**

1. **Lin SL**, **Castano AP**, **Nowlin BT**, **Lupher MLJ**, **Duffield JS**. Bone Marrow Ly6Chigh Monocytes Are Selectively Recruited to Injured Kidney and Differentiate into Functionally Distinct Populations. *The Journal of Immunology*. 2009; **183**:6733–6743. DOI: 10.4049/jimmunol.0901473.

2. **Schrimpf C**, **Xin C**, **Campanholle G**, **Gill SE**, **Stallcup W**, **Lin S-L**, **Davis GE**, *et al.* Pericyte TIMP3 and ADAMTS1 modulate vascular stability after kidney injury. *J. Am. Soc. Nephrol.* 2012; **23**:868–883.DOI: 10.1681/ASN.2011080851.

3. **Kayala MA**, **Baldi P**. Cyber-T web server: differential analysis of high-throughput data. *Nucleic Acids Res.* 2012; **40**:W553–9.DOI: 10.1093/nar/gks420.

4. **Baldi P**, **Long AD**. A Bayesian framework for the analysis of microarray expression data: regularized t -test and statistical inferences of gene changes. *Bioinformatics*. 2001; **17**:509–519.

5. **Storey JD**, **Tibshirani R**. Statistical significance for genomewide studies. *Proc. Natl. Acad. Sci. U.S.A.* 2003; **100**:9440–9445.DOI: 10.1073/pnas.1530509100.

6. **Saeed AI**, **Sharov V**, **White J**, **Li J**, **Liang W**, **Bhagabati N**, **Braisted J**, *et al.* TM4: a free, open-source system for microarray data management and analysis. *BioTechniques*. 2003; **34**:374–378.

7. **Zhang B**, **Kirov S**, **Snoddy J**. WebGestalt: an integrated system for exploring gene sets in various biological contexts. *Nucleic Acids Res.* 2005; **33**:W741–8.DOI: 10.1093/nar/gki475.

8. **Ashburner M**, **Ball CA**, **Blake JA**, **Botstein D**, **Butler H**, **Cherry JM**, **Davis AP**, *et al.* Gene ontology: tool for the unification of biology. The Gene Ontology Consortium. *Nat. Genet.* 2000; **25**:25–29.DOI: 10.1038/75556.

9. **Sanderson S**, **Shastri N**. LacZ inducible, antigen/MHC-specific T cell hybrids. *Int Immunol*. 1994; **6**:369–376.

10. **Hamerman JA**, **Jarjoura JR**, **Humphrey MB**, **Nakamura MC**, **Seaman WE**, **Lanier LL**. Cutting edge: inhibition of TLR and FcR responses in macrophages by triggering receptor expressed on myeloid cells (TREM)-2 and DAP12. *J. Immunol.* 2006; **177**:2051–2055.

11. **Daws MR**, **Sullam PM**, **Niemi EC**, **Chen TT**, **Tchao NK**, **Seaman WE**. Pattern recognition by TREM-2: binding of anionic ligands. *J. Immunol.* 2003; **171**:594–599.

12. **Hsieh CL**, **Koike M**, **Spusta SC**, **Niemi EC**, **Yenari M**, **Nakamura MC**, **Seaman WE**. A role for TREM2 ligands in the phagocytosis of apoptotic neuronal cells by microglia. *Journal of Neurochemistry*. 2009; **109**:1144–1156. DOI: 10.1111/j.1471-4159.2009.06042.x.

**SI Table**

**Table 1. Quantitative PCR from kidney tissue day 5 after U-IRI injury.**

|  | **SHAM** | | **IRI** | |
| --- | --- | --- | --- | --- |
|  | **WT** | ***Dap12-/-*** | **WT** | ***Dap12-/-*** |
| Mip2 | 1±0.3 | 0.9±0.4 | 3.3±0.3 | 3.3±0.4 |
| Trem1 | 1±0.4 | 0.6 ±0.1 | 8.5±0.7 | 10.6±1.9 |
| Il-1β | 1±0.2 | 1.3±0.6 | 4.7±0.7 | 5±1 |
| Ccr2 | 1±0.1 | 0.9±0.1 | 2.5±0.1 | 2.5±0.2 |
| Il-10 | 1±0.1 | 1.2±0.47 | 4.6±0.9 | 4.9±0.2 |
| Tnf-α | 1±0.1 | 1.3±0.47 | 8.2±0.5 | 10.4±1.7 |

**Figure S1. Ly6C Macrophage subpopulations purified from UUO kidney. (A)** Representative plots of total kidney cells from single cell preparation 5 days after UUO were selected for viability and singularity by initial forward and side scatter gates. **(B)** Ly6G+ and NK1.1+ cells were negative gated to exclude neutrophils and NK cells. The different macrophage subpopulation were sorted by gating populations of CD11b+ cells with three levels of Ly6C expression: Ly6C^high^, Ly6C^int^, and Ly6^low^.

**Figure S2. Transcriptional analysis of activated macrophages in sterile kidney injury.** Clustered profiles of 63 differentially expressed genes between Ly6C^+^ (Ly6C^high^ and Ly6C^int^, n = 3/group) and Ly6C^low^ (n = 2) macrophages depicted using a heatmap. Note the progressive decline in *Trem1* expression levels across Ly6C^high^, Ly6C^int^, and Ly6C^low^ sub-populations. Gene Ontology relational representation of highly enriched functional modules corresponding to differentially expressed genes between Ly6C^+^ and Ly6C^low^ macrophages. Prominent processes include immune response, migration, chemotaxis, and cytokine binding and activity.

**Figure S3. Temperature sensitive kidney DAMPs activate macrophages ex vivo.**

**(A)** Q-PCR for *Il-1β* in BMDMφ primed with IFNγ (0, 250 or 500U/ml) for 8 hours, washed, and further stimulated with kidney DAMPs for 12 hours. **(B)** Coomassie blue stained SDS PAGE of crude preparation of soluble extracellular factors from normal (control) and disease kidney (kidney DAMPs). **(C)** Western blotting showing HMGB1 expression in soluble extracellular factors from control and kidney DAMPs. **(D)** Q-PCRs from BMDMφs treated with DAMPs for 16h showing the effect of temperature changes on kidney DAMP activity. **(E)** Q-PCR showing the effect of kidney DAMPs digestion for 16h with Trypsin (1:20 w/w ratio) or Pronase (1:50 w/w ratio) prior application to BMDMφ for 16h. (* P < 0.05, n= 5-7/group, 3 independent experiments; ns, p is not significant).

**Figure S4. TREM-1 pathway is important for BMDMφ activation by LPS *in vitro*, but dispensable for activation by kidney DAMPs.** **(A)** Schema of TREM1-Fc fusion protein and Western blot of purified TREM1-Fc, detected by anti-TREM-1 antibodies. **(B-C)** Q-PCR for *Il-1β* in BMDMφs stimulated with LPS and treated with **(B)** TREM1-Fc or **(C)** anti-TREM-1 antibodies. **(D)** Q-PCR for *Il-1β* in BMDMφ pre-incubated with kidney DAMPs for 8h to induce TREM-1 expression, followed by kidney DAMPs in the presence of anti-TREM-1 antibodies or TREM1-Fc for 16h further. **(E)** Q-PCR showing BMDMφ response to DAMPs for 16h that were pre-adsorbed by hIgG or TREM1-Fc coated protein-A beads. **(F-G)** Colorimetric assay reporting Lacz activity in BWZ-Lacz reporter cells expressing TREM1-DAP12 chimera protein stimulated with kidney DAMPs for 16h in wells **(F)** pre-coated with kidney DAMPs or **(G)** in suspension (anti-TREM-1 antibodies are positive control). (n=3-5/group, 3 independent experiments; *P<0.05).

**Figure S5. Treatment with soluble TREM1-Fc does not prevent macrophage activation, injury and fibrosis in UUO model of sterile kidney injury.** Mice were subjected to unilateral ureter obstruction (UUO) and treated daily with 40μg/mouse of TREM1-Fc or hIgG, as control. **(A)** Q-PCR for different inflammatory transcripts (left) or pro-fibrotic transcripts, Collagen1a1 *(Col1a1)* and alpha smooth muscle actin (*Acta2*), from whole kidney day 5 after UUO. **(B)** Representative images (left) and quantitative graphs (right) showing + F4/80 cells (green), + αSMA (red) or collagen deposition (Sirius Red staining) day 5 after UUO. (* P < 0.05, n= 5-7/group, 3 independent experiments; Bar marker=50µm; Q-PCR data were normalized to sham+higG control).

**Figure S6. The TLR2/4/MyD88 pathway is dispensable in the UUO model of sterile kidney injury. (A,C,E)** Q-PCR for different inflammatory molecules, pro-fibrotic transcripts, collagen1a1 (*col1a1*) and alpha smooth muscle actin (*Acta2*), and the tubule injury marker, kidney injury molecule-1 (*Kim-1*) from whole kidney day 5 after UUO in **(A)** *Myd88^-/-^,* **(C)** *Tlr2-4^-/-^*, and mice lacking MyD88 only in myeloid cells lineage, **(E)** *Csf1R-icre; MyD88^fl/fl^*. **(B,D,F)** Graphs showing quantification of fluorescent images for + αSMA cells and + F4/80 cells. (* P < 0.05, n= 5-7/group; Q-PCR data were normalized to wild type sham).

**Figure S7. Validation of MyD88 conditional ablation in myeloid cells expressing Csf1R.** *Csf1R-iCre* mice were crossed with *Myd88^fl/fl^* to generate *Csf1R-icre; Myd88^fl/fl^* mice, which selectively ablates MyD88 expression in myeloid cells expressing Csf1R. **(A)** Western blot showing basal or LPS-induced MyD88 expression of BMDMφ isolated from *Csf1R-icre; Myd88^+/+^* or *Csf1R-icre; MyD88^fl/fl^*. **(B)** Q-PCR for *Il-1β* expression of BMDMφ from *Csf1R-icre; Myd88^+/+^***,** *Csf1R-icre; MyD88^fl/fl^***,** *Myd88^+/+^* and *Myd88^-/-^* mice stimulated with LPS for 16h. (* P < 0.05, n= 3-5/group; Q-PCR data were normalized to wild type control).
